# Backbone extraction through statistical edge filtering: A comparative study

**DOI:** 10.1371/journal.pone.0316141

**Published:** 2025-01-03

**Authors:** Ali Yassin, Hocine Cherifi, Hamida Seba, Olivier Togni

**Affiliations:** 1 LIB, Université de Bourgogne, Franche-Comté, Dijon, France; 2 ICB UMR 6303 CNRS - Univ, Bourgogne - Franche-Comté, Dijon, France; 3 UCBL, CNRS, INSA Lyon, LIRIS, UMR5205, Univ Lyon, Villeurbanne, France; Scuola IMT Alti Studi Lucca, ITALY

## Abstract

The backbone extraction process is pivotal in expediting analysis and enhancing visualization in network applications. This study systematically compares seven influential statistical hypothesis-testing backbone edge filtering methods (Disparity Filter (DF), Polya Urn Filter (PF), Marginal Likelihood Filter (MLF), Noise Corrected (NC), Enhanced Configuration Model Filter (ECM), Global Statistical Significance Filter (GloSS), and Locally Adaptive Network Sparsification Filter (LANS)) across diverse networks. A similarity analysis reveals that backbones extracted with the ECM and DF filters exhibit minimal overlap with backbones derived from their alternatives. Interestingly, ordering the other methods from GloSS to NC, PF, LANS, and MLF, we observe that each method’s output encapsulates the backbone of the previous one. Correlation analysis between edge features (weight, degree, betweenness) and the test significance level reveals that the DF and LANS filters favor high-weighted edges while ECM assigns them lower significance to edges with high degrees. Furthermore, the results suggest a limited influence of the edge betweenness on the filtering process. The backbones global properties analysis (edge fraction, node fraction, weight fraction, weight entropy, reachability, number of components, and transitivity) identifies three typical behavior types for each property. Notably, the LANS filter preserves all nodes and weight entropy. In contrast, DF, PF, ECM, and GloSS significantly reduce network size. The MLF, NC, and ECM filters preserve network connectivity and weight entropy. Distribution analysis highlights the PU filter’s ability to capture the original weight distribution. NC filter closely exhibits a similar capability. NC and MLF filters excel for degree distribution. These insights offer valuable guidance for selecting appropriate backbone extraction methods based on specific properties.

## Introduction

In recent decades, the analysis of complex systems has found a valuable tool in networks [1]. Networks represent these intricate systems by linking nodes through edges, effectively depicting their interactions. This analysis serves diverse purposes, including community detection [2, 3], identification of influential nodes [4], and understanding network formation [5]. When we gauge the intensity (strength, amount, similarity, etc.) of node interactions, we can assign numerical weights to edges, resulting in what we term weighted networks [6]. Alongside the richness of information that more extensive networks offer, the sparsity principle proves vital for the computational efficiency of many network algorithms. Numerous network analysis and visualization algorithms operate under the assumption that networks possess a certain level of sparsity. One straightforward approach to downsizing a network is setting a threshold on edge weights. Yet, this approach disrupts the natural diversity of edge weight distribution. Researchers have developed various techniques to extract network backbones, effectively diminishing the overall network size while preserving its fundamental characteristics. These techniques fall into two categories: structural and statistical methods.

Structural techniques [7–11] highlight important nodes or edges by targeting network properties. They extract specific sub-structures from the network. For instance, the minimum spanning tree (MST) extracts the subset of edges of the graph that connects all nodes with the minimum total edge weight.

Statistical techniques actively evaluate edge significance within networks. These methods employ statistical tests to determine the relevance of each edge, discarding those that lack significance. Two primary categories exist for statistical filtering techniques, distinguished by the type of statistical test utilized. The initial category employs hypothesis testing, where a null model outlines weight distribution. Observed edge weights are then contrasted with this null model, yielding a calculated p-value. This p-value quantifies the deviation of observed weights from the null model. Within this group, the Disparity filter [12], Polya Urn filter [13], Noise Corrected Filter [14], Marginal Likelihood filter [15], and the ECM filter [16] are situated. The second category relies on the network’s empirical weight distribution to ascertain edge significance. Here, filtering techniques generate p-values by comparing edge weights against the observed weight distribution. The GloSS filter [17] and LANS filter [18] fit into this category.

Next, you can set a significance level and filter out edges with p-values below this level. This significance level reflects the chance of wrongly rejecting the null hypothesis. A prior investigation [19] scrutinized six backbone extraction techniques within the Southeast Asian intercity air transport network. This study includes five structural approaches (global weight thresholding, k-core decomposition, minimum spanning tree, primary linkage analysis, multiple linkage analysis) and one statistical approach (disparity filter) [12, 20–23]. The researchers assessed the extracted backbones regarding geographical and topological structures, highlighting each technique’s potential for diverse transport research applications. For instance, the study recommended k-core decomposition for analyzing the best-connected core in multiplex networks. Primary linkage analysis proved advantageous for dissecting functional/nodal regions and spotlighting hub-and-spoke configurations. Finally, they recommend the Disparity Filter to analyze large-scale networks’ overall topological and spatial information and potentially uncover hidden structures.

Neal et al. [24] compared five statistical backbone extraction techniques designed mainly for bipartite projections: fixed fill model, fixed row model, fixed column model, fixed degree sequence model, and stochastic degree sequence model [25–27]. They used synthetic bipartite networks and two real networks: the Globalization and World Cities (GaWC) and a co-authorship network. The comparative evaluation concerns accuracy, speed, statistical power, similarity, and community structure. Although there is no single winner, they recommend the stochastic degree sequence model for extracting the backbone of most bipartite projections.

Gomes Ferreira et al. [28] proposed a four-step principled methodology for comparing and selecting the most appropriate backbone extraction method given a phenomenon of interest. They validated their approach using two case studies: online discussions on Instagram and coordinated behavior in WhatsApp groups. Results show that each method can produce very different backbones, underlying that choosing an adequate method is of utmost importance to reveal valuable knowledge about the particular phenomenon under investigation.

In a previous study [29], we conducted a comparative assessment of seven statistical techniques for extracting network backbones [12–18] within the USA’s weighted air transportation network. This evaluation was centered on the airlines’ business models. The comparison of the extracted backbones encompassed factors such as component count, size, the proportion of airport types, edge types, and the weights retained by each method. The findings reveal that the Enhanced Configuration Model (ECM) Filter primarily uncovers the infrastructure interlinking regional spoke airports. Conversely, the other filters (Disparity, Polya Urn, Marginal Likelihood, Noise Corrected, Global Statistical Significance (GloSS), Locally Adaptive Network Sparsification (LANS)) highlight the hub-and-spoke framework commonly employed by airlines.

In a preliminary study [30, 31], we explored statistical techniques for extracting network backbones within the weighted World Air Transportation network, which boasts more diversity than the US counterpart. The investigation incorporated seven statistical backbone filtering methods [12–18]. Our findings indicate that the Marginal Likelihood Filter, Disparity Filter, and LANS Filter assign greater importance to high-weight edges. Conversely, the remaining techniques accentuate both smaller and high-weighted edges. We demonstrate that filters based on the binomial distribution, such as the Marginal Likelihood and Noise Corrected filters, maintain a substantial proportion of links and nodes within this case study. However, apart from the ECM Filter, other filters display a more aggressive inclination toward edge removal when dealing with accepted significance levels *α* ≤ 0.05.

Prior research on the comparative analysis of statistical backbone extraction techniques in simple weighted networks has been notably limited. While preliminary studies exist, a comprehensive and systematic comparative analysis has yet to be undertaken. This study fills this void, offering practical implications across various domains.

We address a critical aspect of network analysis by focusing solely on statistical filtering techniques for comparison. These techniques offer adaptability and fine-tuning through significance level adjustments, making them pivotal in real-world applications. This study maintains a precise and targeted examination of the more versatile statistical approaches by intentionally excluding structural filtering techniques.

Extending beyond the initial research [30], this study’s expansion to encompass a diverse range of networks from different domains adds a layer of practicality. The variation in network sizes and fields enhances the findings’ generalizability, catering to a broader range of real-world scenarios.

Adopting various evaluation measures aligns with a comprehensive approach to assessing the effectiveness of the techniques. This thorough evaluation not only aids in better understanding the techniques’ behaviors but also provides a more nuanced perspective for researchers and practitioners to make informed decisions.

Furthermore, incorporating measures from our field and insights from a well-regarded study [32, 33] amplifies the study’s robustness. This hybrid approach reflects best practices and introduces fresh perspectives that can lead to innovative insights.

By dissecting filtering technique similarities, exploring backbone edge characteristics, and analyzing global backbone properties, this study uncovers a wealth of information crucial for deciphering the intricate nuances of network analysis.

In the forthcoming sections, we present a well-structured examination of the filtering techniques and their impact on network backbones. Our study unfolds as follows:

- Similarity Analysis: We commence by scrutinizing the similarities between filtering techniques, employing the Spearman Rank Coefficient and Overlap Coefficient to shed light on their comparative behaviors.
- Backbone Edge Characteristics: Subsequently, we delve into the intricate characteristics of backbone edges. It involves investigating the complex relationships connecting local network properties with edge p-values. This analysis’s pivotal components are edge weight, edge betweenness, and edge degree.
- Comparison of Global Backbone Properties: Our study takes a holistic perspective, comparing global backbone properties across filtering techniques for a commonly employed significance level, *α* = 0.05, with and without multiple testing corrections. We explore variations in essential attributes such as edge proportions, nodes, and weights, weight entropy, reachability, number of components, and transitivity.
- Comparison of Backbone Distributions: We take a deep dive into comparing weight and degree distributions using a two-sample Kolmogorov-Smirnov test (KS test) between the backbones and the original network distributions.

## The statistical backbone extraction methods

The Statistical backbone extraction methods can be classified into two types based on their operational principles: model-based and empirical-based methods.

### The model-based methods

The model-based methods establish a null model to elucidate the generation of edge weights. Then, they compute p-values by comparing the observed weights with those generated by the null model.

#### Disparity Filter (DF) [12]

Assumes that the normalized weights of a node’s edges follow a uniform distribution. It compares observed normalized edge weights to this null model to filter out edges at a desired significance level. This naïve approach is interesting when the underlying distribution of edge weights is unknown, or the generation process of edge weights is unknown. Using a uniform distribution as the null model provides a straightforward approach to assessing the significance of edges in a network. Note that in this study, we then select the minimum p-value for an edge between the two computed probabilities.

#### Polya Urn Filter (PF) [13]

Assumes that edge weights result from an aggregation process of nodes interacting over time. It defines a null model for each edge from the viewpoint of its incident nodes. It employs a Pólya process to calculate the probability of a node distributing its strength randomly. This method is particularly useful for capturing the dynamic nature of interactions in a network, where edge weights may evolve over time. Considering the aggregation process and incorporating a reinforcement parameter offers a nuanced approach to edge significance assessment, allowing for a more tailored analysis of network dynamics. Note that in this study, we select the minimum p-value for an edge between the two computed probabilities.

#### Marginal Likelihood Filter (MLF) [15]

Assumes the likelihood of an edge forming between two nodes is influenced by their respective degrees. In other words, high-degree nodes are more likely to be connected. This probabilistic process is often modeled using a binomial distribution, where the probability of an edge between two nodes depends on their degrees relative to the total degrees in the graph. The null model establishes a baseline by simulating a random ensemble of graphs that share certain characteristics with the observed graph. Specifically, it maintains two key attributes: the total weight of the graph and its degree sequence on average.

#### Noise Corrected Filter (NC) [14]

Assumes that edge weights follow a binomial distribution akin to the Marginal Likelihood Filter. However, it adopts a Bayesian approach to estimate the probability of observing a weight connecting two nodes. This Bayesian framework facilitates the generation of posterior variances for all edges. These posterior variances, in turn, enable the creation of confidence intervals for each edge weight. Ultimately, an edge is removed if its weight falls below a threshold of Δ standard deviations stronger than the expected value, where Δ presents the sole parameter of the algorithm. It also provides a direct approximation through Binomial distribution similar to the Marginal Likelihood Filter. In this paper, we use the approximation method.

#### Enhanced Configuration Model Filter (ECM) [16]

It improves upon the null model used in the Marginal Likelihood Filter by incorporating constraints not only on the strength sequence but also on the degree sequence. It achieves this by leveraging the Enhanced Configuration Model for network reconstruction. This model constructs a null model by creating a maximum-entropy ensemble of weighted networks, ensuring they share the same degree and strength distribution as the real network. Then, it compares the observed edge weights in the real network with the expected edge weights based on the null model.

#### Global Statistical Significance Filter (GloSS) [17]

The null model used to assign p-values to edges involves creating a network with the exact same topology as the original network. However, in this null model, link weights are randomly drawn from the empirical weight distribution of the original network. This approach effectively shuffles the observed link weights over the existing fixed network topology. By doing so, the GloSS method avoids making assumptions about the distribution of weights and instead relies on the empirical distribution derived from the original network data.

### The empirical-based methods

The empirical-based methods do not rely on a predefined null model. Instead, they use the edge weights empirical distribution to compute the probability of observing the given weight.

#### Locally Adaptive Network Sparsification Filter (LANS) [18]

Operates without assuming any specific underlying weight distribution, relying exclusively on empirical data. It employs the empirical cumulative density function to assess the statistical significance of edge weights. Specifically, each incident node of an edge computes the probability of randomly selecting an edge with a weight equal to or greater than the observed weight. In this study, we choose the minimum probability between the two computed probabilities.

Throughout the remainder of the paper, we use the following abbreviation: MLF for Marginal Likelihood Filter, DF for Disparity Filter, LANS for Local Adaptive Network Sparsification, PF for Polya Urn Filter, NC for Noise Corrected Filter, GloSS for Global Statistical Significance Filter, and ECM for Enhanced Configuration Model Filter.

## Filtering strategies and multiple testing corrections

In traditional single-hypothesis testing, filtration involves computing p-values based on the model and filtering p-values below a predetermined significance level, usually denoted as *α*. However, applying such a simplistic approach becomes problematic when dealing with multiple-hypothesis testing. The number of hypotheses being tested increases the likelihood of false positives, necessitating the implementation of multiple testing corrections to control this inflated error rate.

One widely used method for such corrections is the Bonferroni method [34], which adjusts the significance level to account for the increased number of tests performed. Another notable approach is the False Discovery Rate (FDR) estimation, particularly the Benjamini-Hochberg (FDR-BH) method [35], which offers a more nuanced adjustment by controlling the expected proportion of false discoveries among all discoveries made.

**Fig 1:**
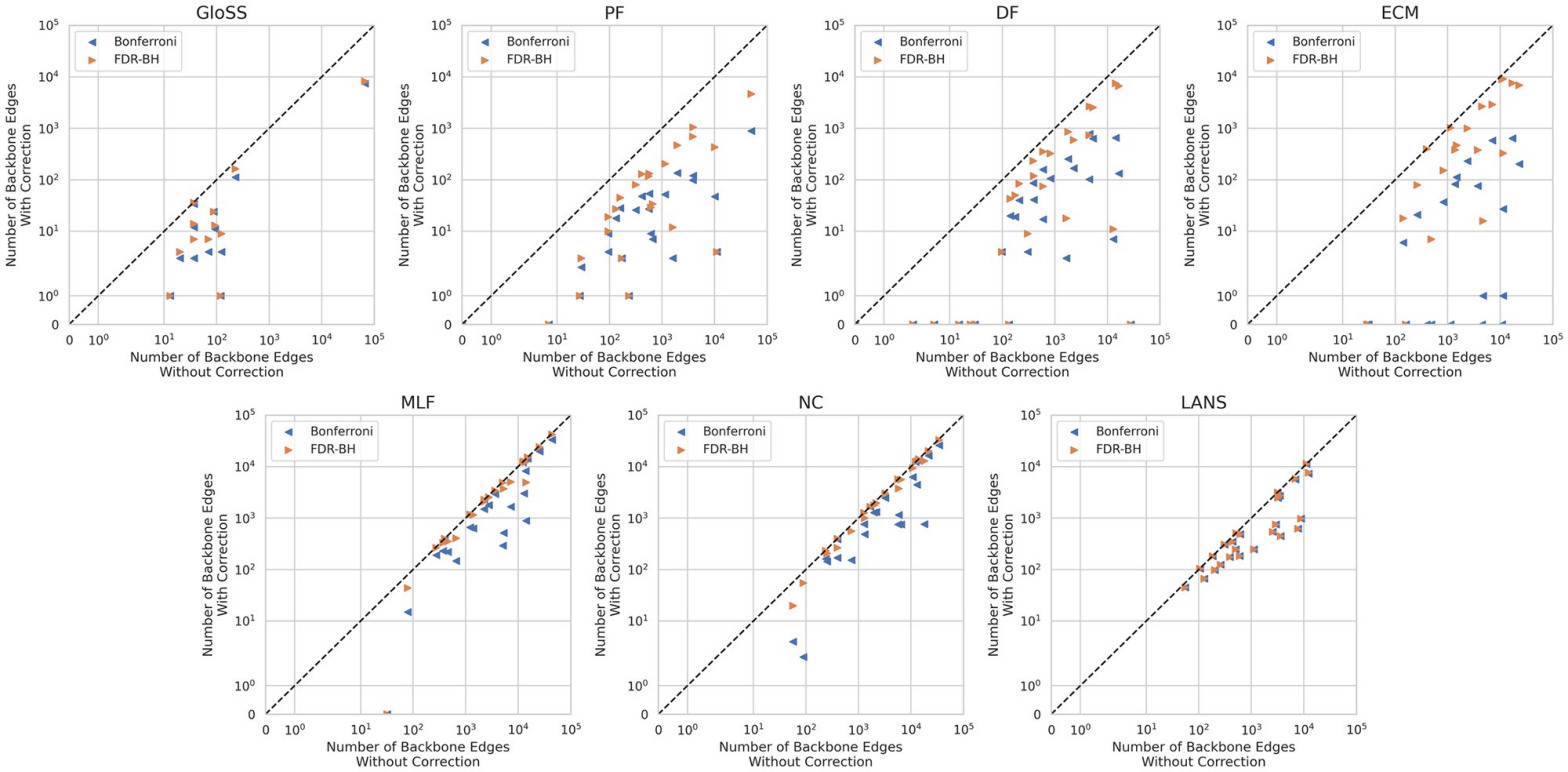
The number of edges in the backbones extracted without multiple testing corrections for a significance level of *alpha* = 0.05 against the number of edges in the backbones extracted with multiple testing corrections strategies for the same significance level.

We apply these correction methods using a confidence level of *α* = 0.05 and compare the number of retained edges across all networks, as depicted in Fig 1 on a log-log scale. Two distinct behaviors emerge:

The GloSS, Polya Urn (PF), Disparity (DF), and ECM filters illustrate the first one reported in the top row. Notably, a substantial drop in the number of backbone edges occurs upon applying corrections, especially with the Bonferroni correction. Additionally, in many networks, the edge count decreases to fewer than 10, particularly in the GloSS, Polya Urn (PF), and Disparity (DF) filters after correction. This suggests a high likelihood of false positives in these methods, mitigated by corrections, albeit resulting in only a few remaining significant edges that can be easily counted manually.

The second behavior reported in the bottom row concerns the Marginal Likelihood (MLF), Noise Corrected (NC), and LANS filters. The number of backbone edges for the MLF and NC filters remains nearly unchanged when employing the FDR-BH correction. However, a slight decrease is observed when using the Bonferroni correction. Conversely, the Bonferroni and FDR-BH corrections yield the same number of backbone edges for the LANS filter. It indicates a low likelihood of false positives in these methods, as corrections have minimal impact on the number of backbone edges.

It’s important to note that although filtering relies on p-values, remember that it does not measure the relevance of an event and that care must be taken when using it for edge filtering.

## Networks overview

**Fig 2:**
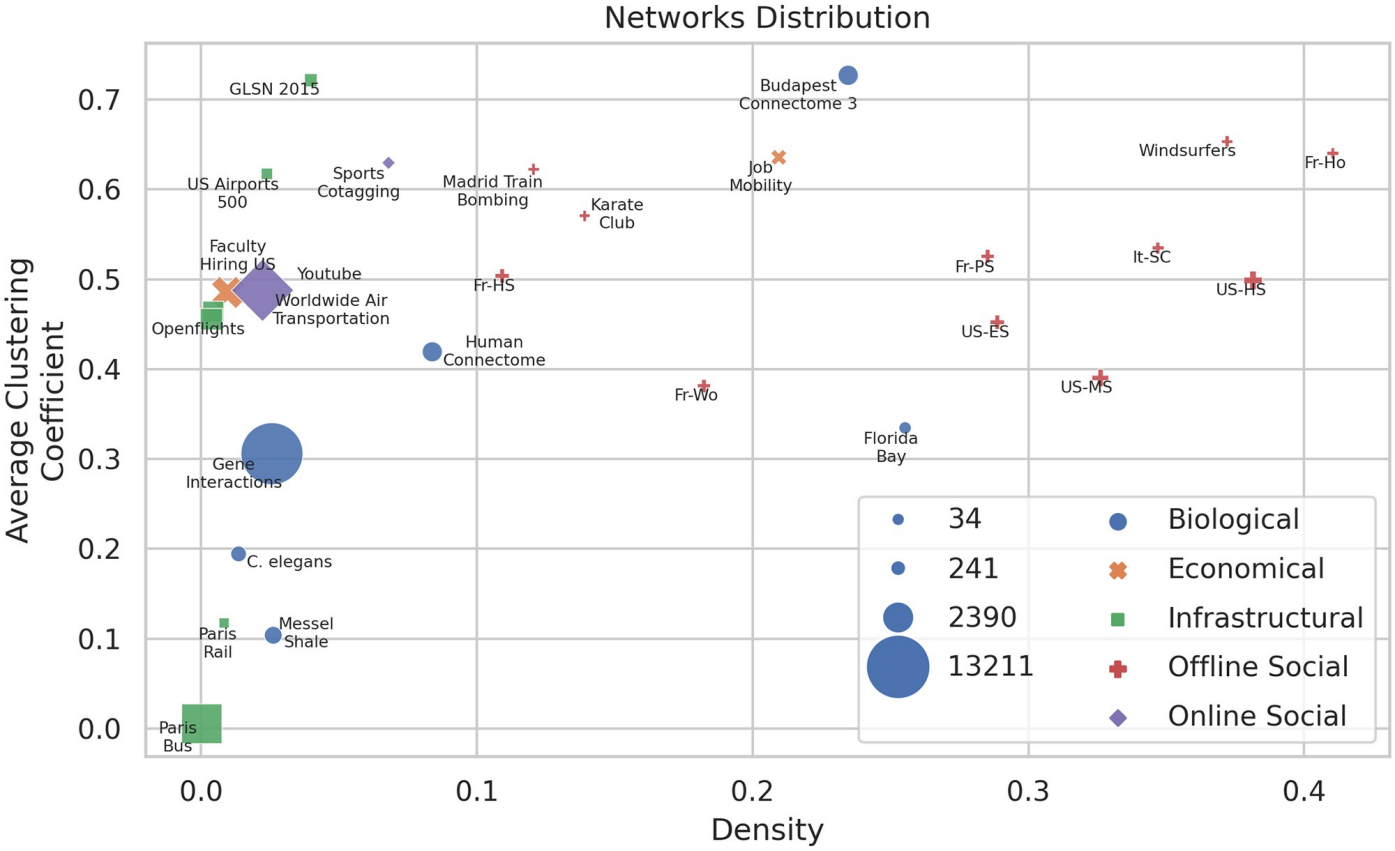
A scatter plot illustrating the distribution of networks based on density and average clustering coefficient. Marker sizes represent the number of nodes in each network.

The data used in the experiments consists of 27 real-world networks across various domains. Fig 2 illustrates the network diversity based on density and average clustering coefficient in a scatter plot. Marker sizes represent the number of nodes in each network. We refer to the S1 Appendix for a detailed description of each network.

### Biological networks

Includes networks such as the Budapest Connectome [36, 37], Human Connectome [38], C. elegans [39], Gene Interactions [40], Messel Shale [41], and Florida Bay [42] networks, each representing different biological systems such as brain connectivity, genetic interactions, and ecological food webs.

### Economic networks

Consists of the Faculty Hiring US [43] and Job Mobility (https://www.michelecoscia.com/?page_id=312) networks, which depict patterns of academic hiring and job transitions, respectively.

### Infrastructural networks

Encompass networks like Worldwide Air Transportation [44, 45], Openflights [46], US Airports 500 [47], Paris Bus [48], Paris Rail [48], and GLSN 2015 [49], representing various infrastructural systems such as air transportation, public transportation, and international shipping routes.

### Offline social networks

Comprise networks such as Karate Club [50], Madrid Train Bombing Terrorists [51], Windsurfers [52], American High School [53], American Middle School [54], American Elementary School [54], French Primary School [55], French High School [55], Workplace, ACM Hypertext 2009 Scientific Conference [55], and Geriatric Ward of French Hospital [55] networks, depicting social interactions in different offline contexts including schools, workplaces, and events.

### Online social networks

Include networks like Sports Cotagging [56] and various Youtube [57] networks, representing interactions among users or topics in online platforms.

## Exploring technique similarities

This section aims to clarify the similarities among backbone filtering techniques using two complementary measures: the Spearman rank correlation coefficient and the Overlap coefficient.

### Edge p-values correlation

Using the Spearman rank correlation, one can assess the relationships between the edge p-values of backbone extraction methods. We compute the edge p-values for each network using the backbone extraction methods. Then, we compute Spearman rank correlation coefficients for each pair of methods for each network. Finally, we aggregate the correlation coefficients by computing the mean and standard deviation across all networks. For the detailed procedure, we refer to Methods.

**Fig 3:**
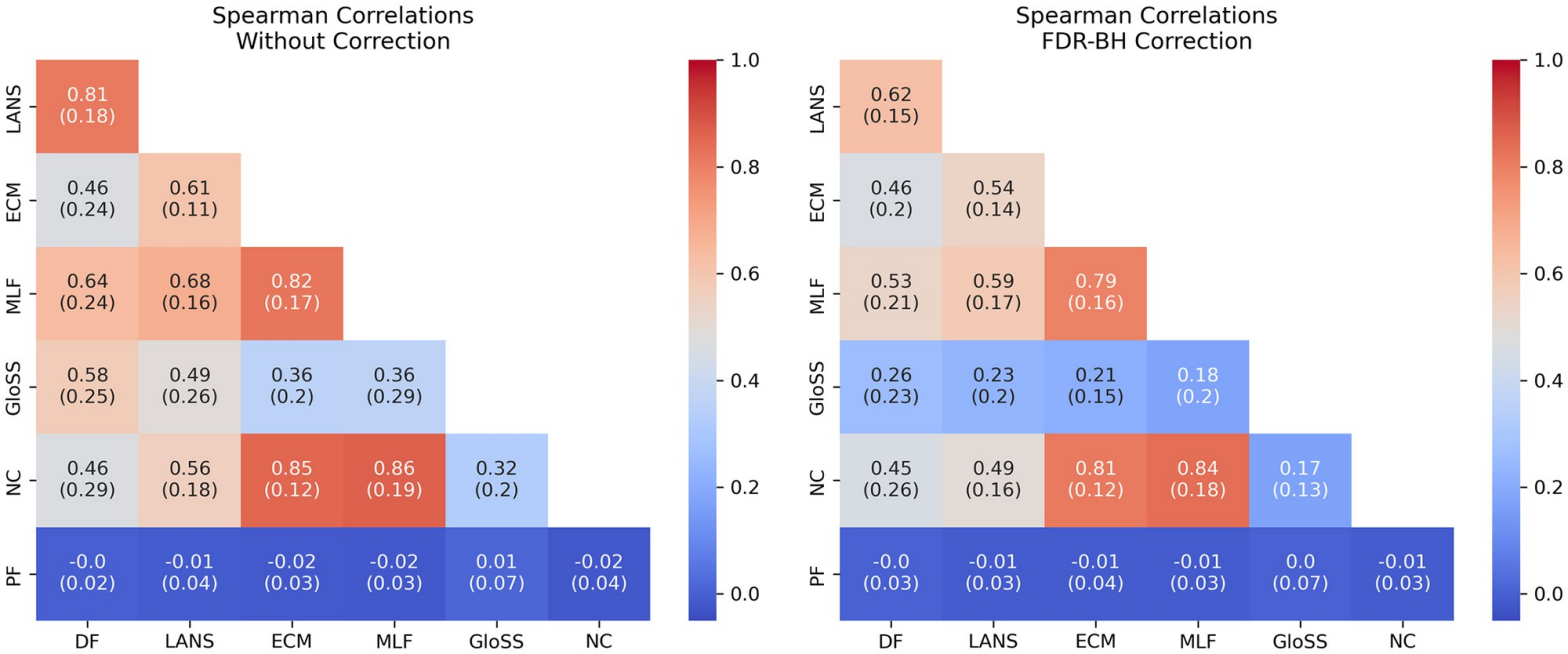
Heatmaps showing the mean Spearman rank correlation of p-values between pairs of backbone filtering techniques across all networks, with and without correction. Techniques include: Marginal Likelihood Filter (MLF), Disparity Filter (DF), Local Adaptive Network Sparsification (LANS), Polya Urn Filter (PF), Noise-Corrected Filter (NC), and Global Statistical Significance Filter (GloSS). Standard deviations are indicated in parentheses.

Fig 3 depicts the mean Spearman rank correlation of p-values (uncorrected and corrected) between pairs of backbone filtering techniques across all networks. The correlation analysis reveals significant associations between specific pairs of backbone filtering techniques. Particularly noteworthy are the strong positive correlations observed between the Disparity Filter and LANS Filter (DF-LANS), as well as among the trio of Enhanced Configuration Model filter (ECM), Marginal Likelihood filter (MLF), and Noise Corrected filter (NC) (ECM-NC, ECM-MLF, MLF-NC). It indicates that when one method ranks an edge higher in a network, the other also ranks it higher.

In Contrast, the Polya Urn Filter stands out due to its lack of correlation with any other technique. This weak relationship may stem from its unique approach compared to its alternative under study.

The other filtering techniques exhibit variable behavior across diverse networks. The inconsistency in rankings across networks suggests that the specific structure of each network notably impacts edge rankings. This emphasizes considering each network’s characteristics when selecting a filtering technique.

Lastly, the consistency demonstrated by the low standard deviation heatmap reinforces the reliability of the observed correlations and behaviors, providing further credibility to the findings presented.

These findings reveal consistent patterns among the initial edge p-values derived from various backbone extraction methods. It’s crucial to note that they are based solely on the raw edge p-values before filtration occurs, offering valuable insights into potential similarities in backbone extraction outcomes across different methodologies.

In the second panel, the correlation values decrease slightly; however, the overall trends remain consistent with the previous results. This indicates that the FDR-BH correction does not significantly impact the observed correlations between the backbone extraction methods.

### Backbone Overlap Coefficient

The focus now shifts to the Overlap Coefficient [58]. Unlike the Spearman Rank Correlation, the Overlap Coefficient centers on edge presence or absence for similarity assessment. It measures the overlap between edge sets derived from different backbones.

The process begins by calculating edge p-values for each network using the backbone extraction methods. Then, backbones are extracted for a significance level of *α* = 0.05 with and without FDR-BH correction. Subsequently, the Overlap Coefficient is computed in each network using the edge sets from each pair of methods’ backbones. The outcomes are aggregated, and each pair’s mean and standard deviation are determined across all networks. This method offers insights into the overlaps between backbone extraction methods across diverse networks. For a detailed procedure, please refer to Methods.

**Fig 4:**
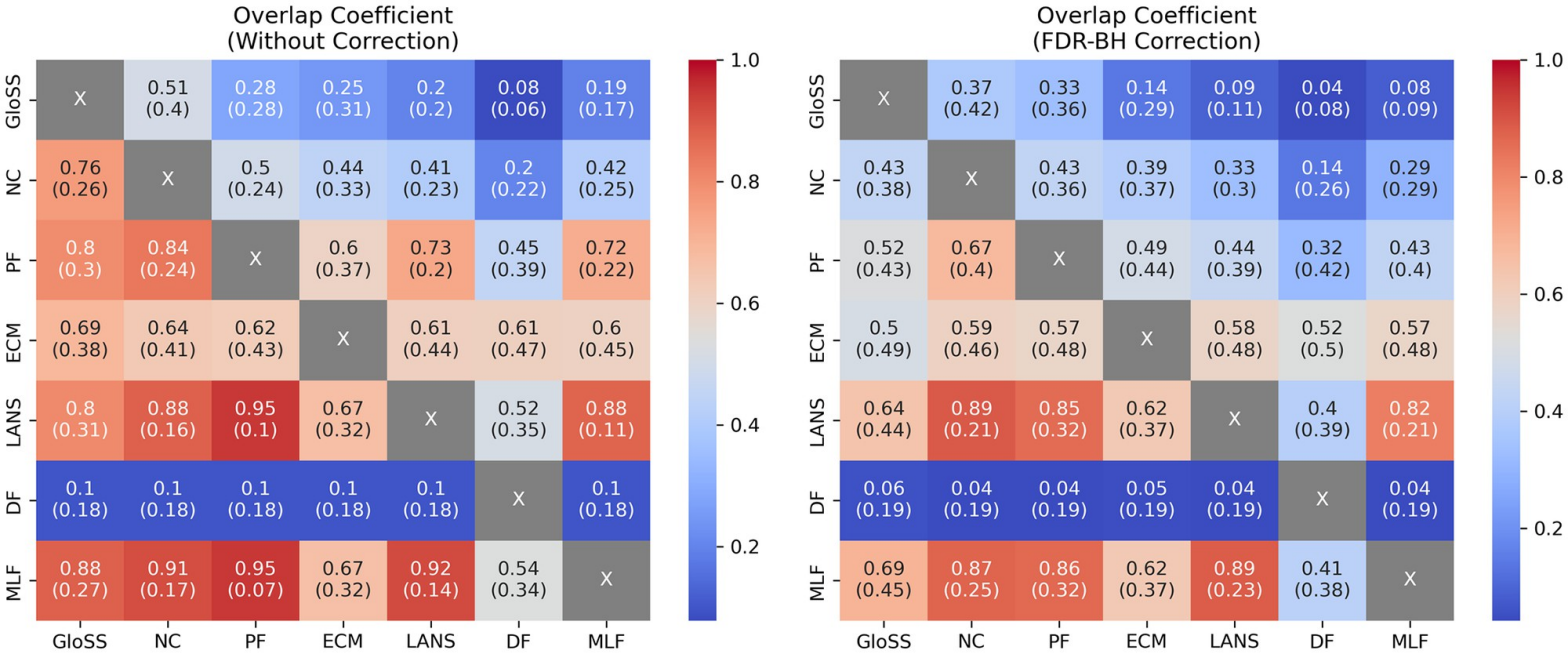
The heatmaps illustrate the mean and standard deviation of the Overlap Coefficient between sets of edges for various backbone pairs extracted without and with FDR-BH correction. As the Overlap Coefficient is calculated relative to each method within the pairs, the resulting heatmap is asymmetrical.

Fig 4 showcases heatmaps illustrating the mean and standard deviation of the Overlap Coefficient between sets of edges for various pairs of backbones, extracted without and with FDR-BH correction.

In the left panel, no correction is applied before extracting the backbones. It shows a hierarchical relationship. It indicates the cumulative inclusion of edges as one progresses from GloSS to Noise Corrected, Poly Urn, LANS, and Marginal Likelihood Backbone (*GloSS* ⊂ *NC* ⊂ *PF* ⊂ *LANS* ⊂ *MLF*). Indeed, on average, the GloSS backbone overlaps by 76% with the Noise Corrected backbone, which overlaps by 91% with the Poly Urn backbone, which overlaps by 95% with the LANS backbone, which finally overlaps by 92% with the Marginal Likelihood backbone. Notably, the standard deviation is relatively low, suggesting the consistency of this hierarchy across all networks.

The backbones overlap, on average, by approximately 65% with the ECM backbones and by 10% with the Disparity backbones. However, the Disparity backbones overlap more with the other backbones. Indeed, they share different fractions of edges with different backbones, at least 8% with the GloSS backbones and at most 60% with the ECM backbones. The high standard deviation of these observations indicates that these findings are not consistent across all networks.

In the right panel, the FDR-BH correction is applied. We observe a change in the hierarchy. The GloSS backbone no longer overlaps with the Noise Corrected backbone, which in turn overlaps less with the Poly Urn backbone. In both cases, the standard deviation is too high to consider this behavior consistent across all networks. Thus, the standing hierarchies with low standard deviations are *PF* ⊂ *LANS* ⊂ *MLF* and *NC* ⊂ *LANS* ⊂ *MLF*.

In summary, while the Spearman rank correlation indicates a monotonic relationship between pairs such as LANS-DF, ECM-NC, NC-MLF, and MLF-ECM, the subsequent experiment demonstrates that these relationships are not consistently reflected in the resulting backbones, except for the NC-MLF pair. In fact, most pairs exhibit low or inconsistent edge overlap between their backbones. However, the experiment highlights a notable exception with the NC-MLF pair, showing a high overlap between Noise Corrected, LANS, and Marginal Likelihood backbones (*NC* ⊂ *LANS* ⊂ *MLF*). Conversely, although Polya Urn and GloSS exhibit the least correlation with other methods, the second experiment reveals their inclusion in other backbones: *GloSS* ⊂ *NC* ⊂ *PF* ⊂ *LANS* ⊂ *MLF*.

Finally, we do not observe complete overlap between the backbones or encounter highly distinct backbones. The most unique backbones identified in this study are derived from the ECM and Disparity (DF) filters. Conversely, the remaining backbones exhibit considerable edge-sharing, aligning with the revealed hierarchy. This proves advantageous in selecting a suitable backbone extraction method. For instance, employing the Marginal Likelihood filter would yield a backbone comprising significant edges that deviate from its null model. Moreover, there is a high probability that these edges also deviate from previous null models in the hierarchy.

## Exploring relationships between filtering techniques and local edge properties

This experiment explores how p-values correlate with local edge properties. It uncovers how edge significance relates to specific network attributes. By investigating these associations, we understand whether specific properties persist after filtering. We compute the Spearman rank correlation between edge p-values and each network property (weight, degree, betweenness). The correlation values for each method across all networks are presented in a boxplot. For the detailed procedure, we refer to Methods.

### Filtering techniques and edge weight correlation

**Fig 5:**
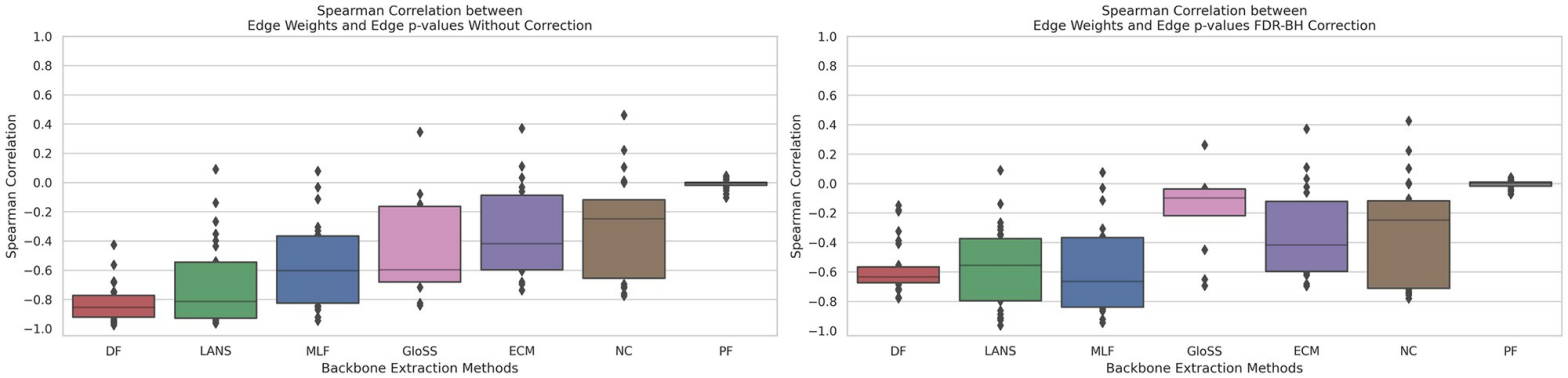
The boxplots of Spearman rank correlation coefficients between edge weights and p-values (uncorrected and corrected) across all networks for various backbone extraction methods. Techniques include: Marginal Likelihood Filter (MLF), Disparity Filter (DF), Local Adaptive Network Sparsification (LANS), Polya Urn Filter (PF), Noise-Corrected Filter (NC), and Global Statistical Significance Filter (GloSS).

The statistical backbone filtering techniques depart from the simplistic edge weights thresholding of the global threshold backbone extraction process. To examine this aspect, we investigate the correlation between p-values and their corresponding edge weights. Fig 5 shows the boxplots of Spearman rank correlation coefficients between edge weights and p-values (uncorrected and corrected) across all networks for various backbone extraction methods.

Our analysis uncovers various behaviors. This diversity underscores the distinct sensitivities of these techniques to network characteristics. Most notably, the observed correlation values tend to be negative, indicating that as the edge weight increases, the p-value decreases, signifying greater significance of the edge.

The mean correlation coefficient of −0.8 between p-values derived from the Disparity Filter (DF) and edge weights underscores a robust negative association, suggesting that the Disparity Filter (DF) prioritizes edges with higher weights. Similarly, the LANS filter exhibits a comparable mean correlation value but with a higher standard deviation and a few outlier networks showing no correlation between the edge p-values and edge weights.

The Polya Urn Filter stands out with its near-zero correlation coefficient and remarkably low standard deviation. It shows that the Polya Urn Filter’s edge selection is unrelated to edge weights, focusing instead on alternative network features.

The mean correlation coefficients for the remaining techniques range from −0.6 to −0.3, indicating moderate correlations with edge weights. This suggests these techniques consider a combination of factors beyond edge weights in their backbone extraction decisions.

These findings underscore the intricate interplay between backbone filtering techniques and edge weights. Opting for global thresholding on the weights tends to favor high edge weights, making the use of statistical backbone extraction methods preferable. While some methods show a strong correlation between edge p-values and weights, like the Disparity filter, they detect more low edge weights than the global threshold method. On the other hand, the Polya Urn filter departs from the global threshold. The other methods fall somewhere between these two extremes. This underscores the importance of taking an informed and context-aware approach when selecting the most appropriate method for a given network, ensuring the extraction of meaningful backbone outcomes.

In the second panel, a significant change is observed in the GloSS filter, where the mean correlation decreases markedly from approximately -0.6 to 0.1 following the FDR-BH correction. This suggests that the correction effectively removes the relationship between edge p-values and edge weights in the GloSS filter. In contrast, no substantial differences are observed in the ECM, NC, and PF filters, which aligns with their previously demonstrated low likelihood of false positives. Finally, the correlation values exhibit a slight decrease in the DF, LANS, and MLF methods, indicating minimal impact from the correction on these approaches.

### Filtering techniques and edge degree correlation

**Fig 6:**
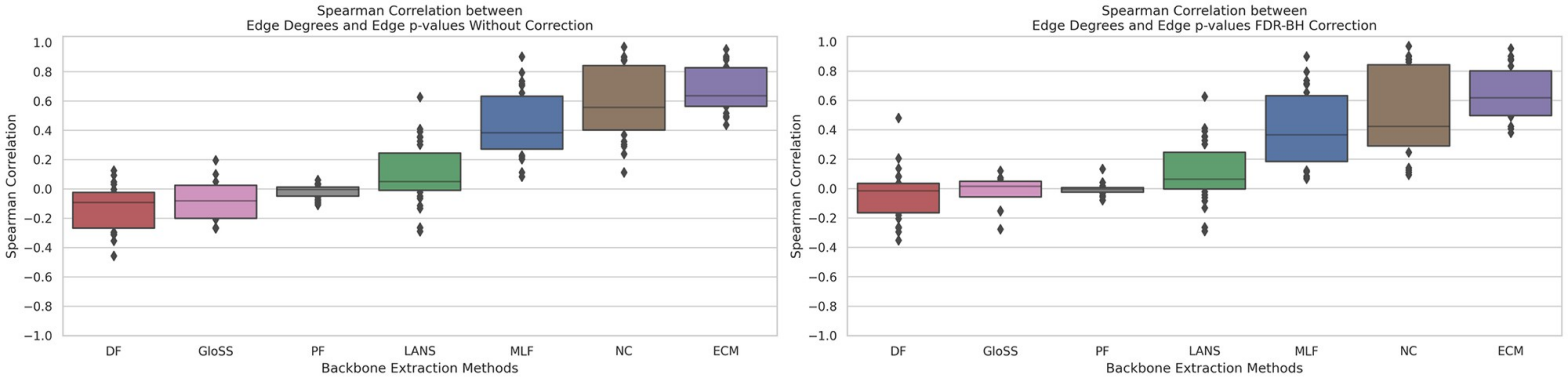
The boxplots of Spearman rank correlation coefficients between edge degrees and p-values (uncorrected and corrected) across all networks for various backbone extraction methods. Techniques include: Marginal Likelihood Filter (MLF), Disparity Filter (DF), Local Adaptive Network Sparsification (LANS), Polya Urn Filter (PF), Noise-Corrected Filter (NC), and Global Statistical Significance Filter (GloSS).

We define the Degree of an edge connecting node *i* with node *j* as the product of the degrees of the connected nodes, represented as *k*(*i*, *j*) = *k*_*i*_*k*_*j*_ [32]. High-degree edges often link network hubs, a common feature in scale-free networks. We calculate the Spearman rank correlation between edge degrees and p-values extracted from diverse backbone filtering techniques across all networks to grasp how backbone filtering techniques handle hub-connected edges. This investigation yields crucial insights into how these techniques navigate the web of hub-connected edges in complex networks. Fig 6 shows boxplots of Spearman rank correlation coefficients between edge degrees and p-values (uncorrected and corrected) across all networks for various backbone extraction methods.

This experiment unveils a range of behaviors exhibited by backbone filtering techniques concerning edges linked to network hubs. This diversity underscores the varied approaches these techniques take in addressing hub-connected edges. Notably, the observed correlation values generally lean towards the positive side, indicating that as the edge weight increases, the p-value also increases, suggesting a decrease in the significance of the edge.

The mean correlation coefficient of 0.6 between p-values derived from the ECM and Noise Corrected (NC) filters and edge weights highlights a positive association, indicating that these methods tend to assign lower values to edges with higher edge degrees within the network, particularly those connecting hubs.

In contrast, the Polya Urn Filter demonstrates an almost zero correlation coefficient between its edge p-values and edge degrees, with a small standard deviation. It suggests that the degrees of connected nodes have minimal influence on the Polya Urn Filter’s edge selection process.

The mean correlation coefficients for other techniques range between −0.1 and 0.4, indicating a slight correlation with edge degrees. These techniques may consider various factors beyond edge degrees in their backbone extraction decisions.

These findings highlight the intricate relationship between backbone filtering techniques and edge degree. This interaction exposes how the ECM filter tends to undervalue connections between hubs, prioritizing connections between low-degree nodes. For example [29], in air transportation networks where airlines often adopt the hub-and-spoke business model, all methods aimed to uncover the hub-and-spoke structure employed by airlines, except for the ECM filter. Instead, the ECM filter focuses on revealing spoke-spoke regional connections within such networks.

Conversely, the Polya Urn filter (PF) preserves a comparable fraction of hub-hub, hub-spoke, and spoke-spoke edges, uncovering both the hub-and-spoke foundation and some regional connections. This underscores the importance of adopting an informed and context-aware approach when selecting the most suitable method for a given network, ensuring meaningful backbone outcomes are extracted.

The second panel reveals consistent trends, showing no significant change in the relationship between edge degrees and p-values after the correction. This indicates that removing false positive edges minimally impacts the observed correlations between edge degrees and the corrected p-values.

### Filtering techniques and edge betweeness correlation

The Betweenness of an edge [59] *e* is the sum of the fraction of all-pairs shortest paths that pass through the edge *e*. It is expressed mathematically as $b\left(e\right)=\sum_{s,t\in N}\frac{\sigma (s,t|e)}{\sigma (s,t)}$, where *N* is the set of nodes, *σ*(*s*, *t*) is the number of shortest (*s*, *t*)-paths, and *σ*(*s*, *t*|*e*) is the number of those paths passing through edge *e*. Filtering the network based on edge betweenness scores involves setting a threshold to retain edges with high betweenness. Thereby extracting the network’s backbone and highlighting essential pathways and connections while removing peripheral ones.

**Fig 7:**
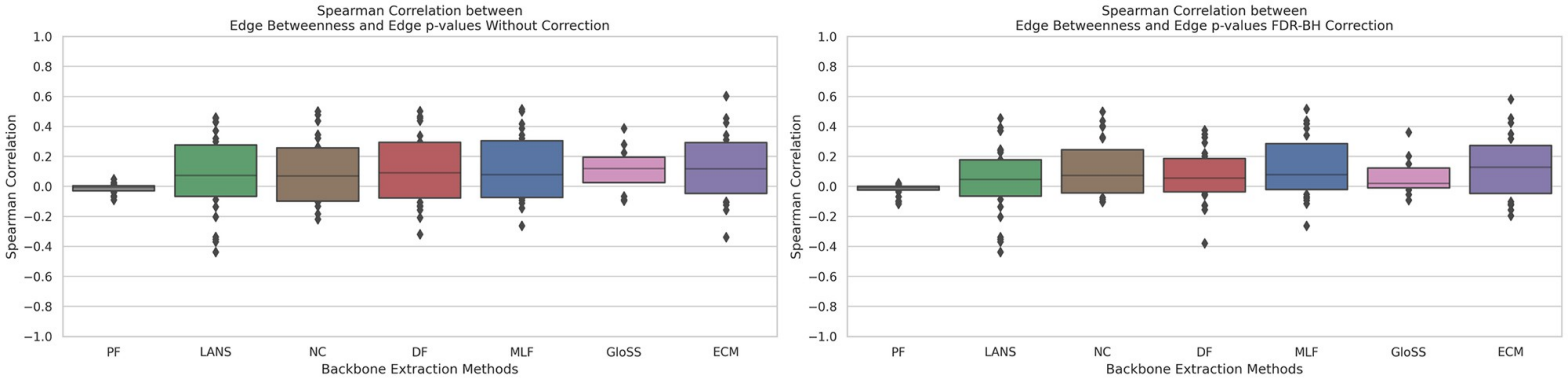
The boxplots of Spearman rank correlation coefficients between edge betweenness and p-values (uncorrected and corrected) across all networks for various backbone extraction methods. Techniques include: Marginal Likelihood Filter (MLF), Disparity Filter (DF), Local Adaptive Network Sparsification (LANS), Polya Urn Filter (PF), Noise-Corrected Filter (NC), and Global Statistical Significance Filter (GloSS).

The box plots in Fig 7 depict Spearman rank correlation coefficients between edge betweenness and p-values (uncorrected and corrected) across all networks for various backbone extraction methods. Despite slight mean correlation coefficients, typically around 0.1, and fluctuating standard deviations, there’s nearly zero correlation between p-values and edge betweenness with the Polya Urn Filter. This result is supported by a very low standard deviation. Overall, these results indicate that backbone filtering techniques, irrespective of differences, show no significant correlation between edge betweenness and p-values. They provide fair scores for both essential pathway edges and peripheral ones, departing from solely prioritizing essential pathways based on edge betweenness.

The second panel reveals consistent trends, showing no significant change in the relationship between edge degrees and p-values after the correction. This indicates that removing false positive edges minimally impacts the observed correlations between edge betweenness and the corrected p-values.

In summary, the findings in this section highlight the importance of certain backbone extraction methods in various use cases. For example, researchers seeking a method akin to the global threshold but operating at a local node level may opt for the Disparity and LANS filters. Conversely, the Polya Urn filter exhibits distinct behavior, deviating significantly from the global threshold.

Moreover, the ECM filter underestimates connections between network hubs, making it suitable for uncovering spoke-and-spoke connections. In contrast, the Polya Urn Filter (PF) preserves a balanced representation of all edge types, making it ideal for identifying both hub-and-spoke structures and spoke-and-spoke connections within complex networks.

Importantly, none of the statistical backbone extraction methods demonstrate a p-value correlation with edge betweenness, indicating their fairness in treating peripheral edges.

## Exploring the backbones global properties

This section explores how various methods shape global network features. Initially, we compute the edge p-values within each network using different backbone extraction methods. Next, we apply the FDR-BH correction. Then, we extract the backbones for a significance level of *α* = 0.05. Following this, we compute the backbone properties with and without correction. Finally, we normalize these properties using the original network values and visualize the results’ Cumulative and Counter Cumulative Distribution Functions. For a detailed explanation of the procedure, please refer to Methods.

### Edge fraction

**Fig 8:**
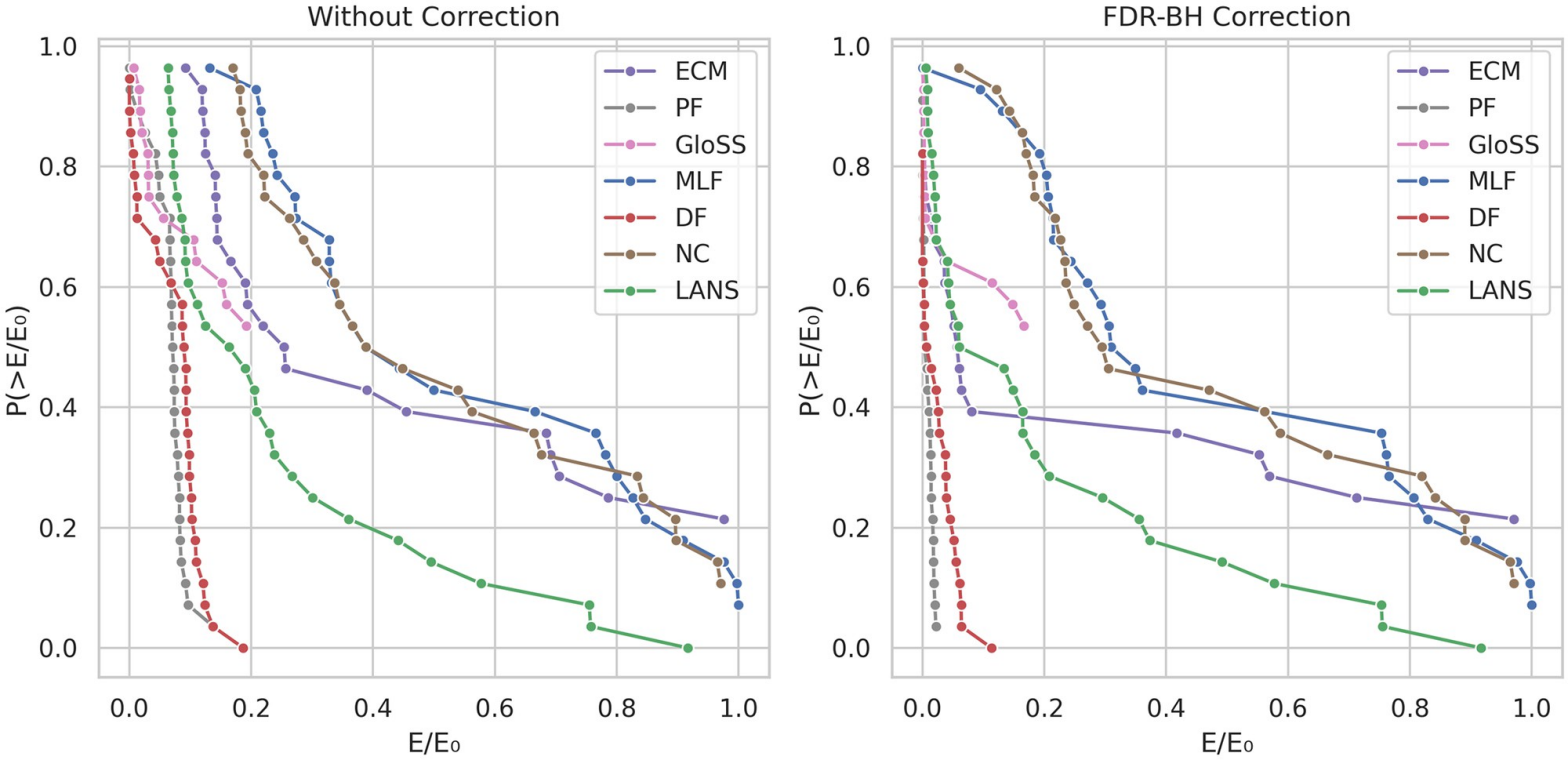
The counter cumulative distribution function of the backbones edge fraction *E*/*E*_0_ across all networks.

The Statistical filtering methods aim to identify significant deviations from a defined null model and filter out the rest. However, some of these filtering models can effectively capture how the weights are generated in the real network, retaining all network edges for the backbone. To examine the performance of these models across a diverse range of networks, we present the counter cumulative distribution function of the edge fraction of the backbones, both without multiple testing corrections and with FDR-BH corrections at a significance level of *α* = 0.05, as shown in Fig 8.

In the left panel, two distinct behaviors appear. The first one concerns the Polya Urn (PF), Disparity (DF), and GloSS filters. For these methods, the curves start with close to zero edge fractions, sharply declining with an edge fraction around 0.1. Furthermore, their tails taper off around 0.2 edge fraction. It indicates that these methods identify a few significant deviations and consistently maintain a fraction of edges less than 20% across all networks.

The second behavior concerns the Noise Corrected (NC), Marginal Likelihood (MLF), ECM, and LANS filters. The curves of these methods initiate around 0.1 and continue to decrease with less steepness. Additionally, their tails extend to values between 0.9 and 1, suggesting that these models effectively capture the network structure, thus struggling to identify significant deviations in many networks.

The right panel reports the results after applying the FDR-BH correction. All methods’ curves, except for the Noise Corrected (NC), sharply decline to almost zero edge fraction. It indicates a high prevalence of false positive edges in these networks. Conversely, while the edge fraction in the Noise Corrected (NC) decreases, it does not reach zero when all edges are removed. This suggests the presence of a low proportion of false positive edges and highlights the robustness of its model.

### Node fraction

**Fig 9:**
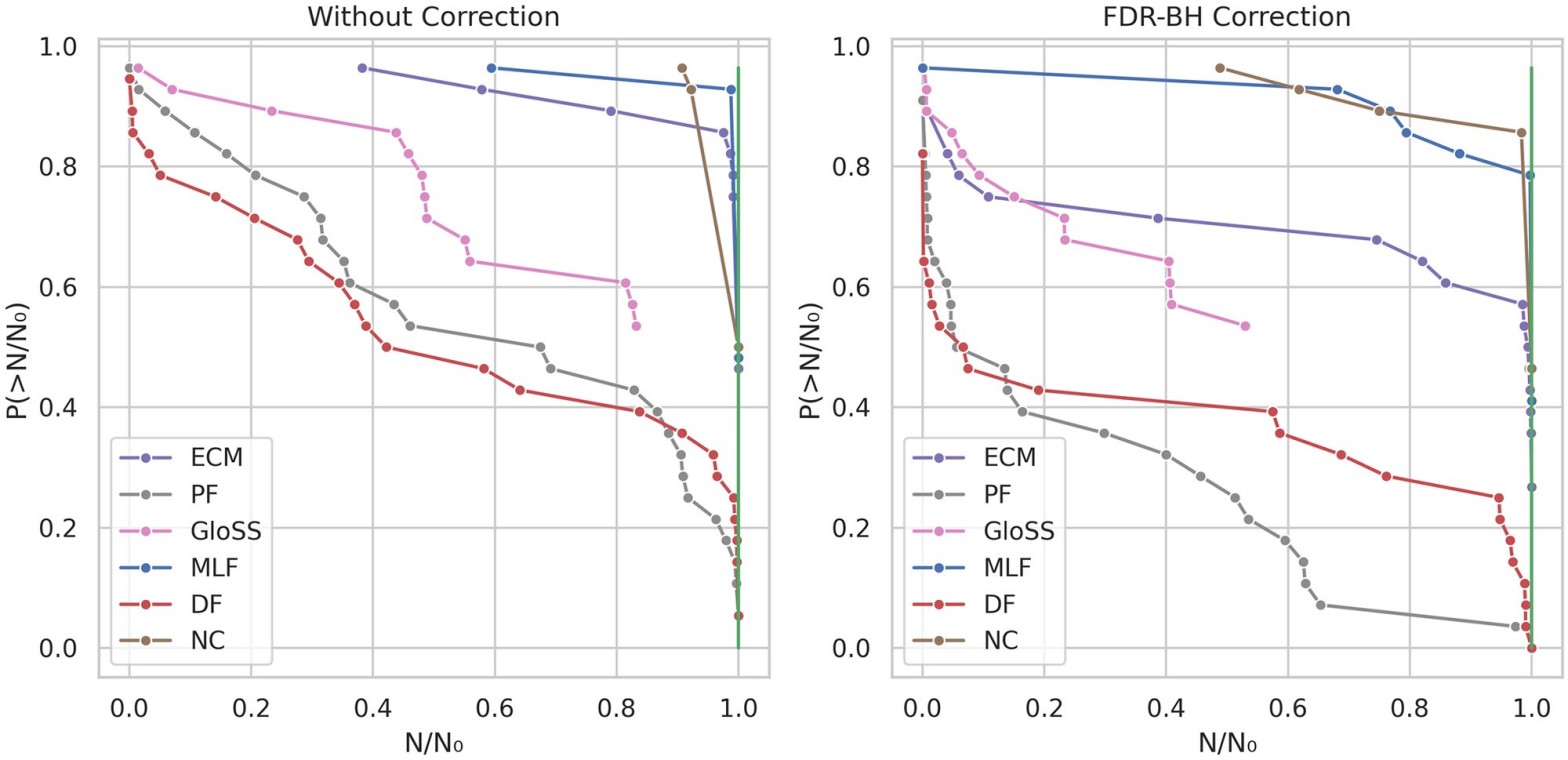
The counter cumulative distribution function of the backbones node fraction *N*/*N*_0_ across all networks.

Preserving nodes is not always an ideal characteristic for a filtering technique. However, maintaining network nodes throughout filtering poses a considerable challenge for many techniques. Therefore, we assess the performance of these models across diverse networks by presenting the counter cumulative distribution function of the node fraction of the backbones, both with and without FDR-BH corrections at a significance level of *α* = 0.05, as depicted in Fig 9.

In the left panel, three distinct behaviors emerge. The First concerns the Polya Urn (PF), Disparity (DF), and GloSS filters. These methods start with minimal node fractions, with their tails tapering off near 1 except for the GloSS curve, which tapers around 0.8. This indicates that the GloSS filter consistently isolates nodes during filtering. While the Disparity (DF) and Polya Urn (PF) filters also isolate numerous nodes, they maintain all nodes in a few networks.

The second behavior is exhibited by the Noise Corrected (NC), Marginal Likelihood (MLF), and ECM filters. Their curves begin at high node fraction values, approximately 0.6 for Marginal Likelihood (MLF) and ECM, and 0.9 for Noise Corrected (NC). Moreover, all their tails extend to reach 1. This suggests that many edges significantly deviating from these methods’ null models are located at the network periphery, explaining their less isolating and more preservative behavior. This aligns with previous findings, indicating a higher correlation between these methods and edge degree than other methods.

The third behavior concerns, the LANS filter. It exhibits a distinct behavior by consistently preserving all the network nodes.

In the right panel, after applying the FDR-BH correction, we observe a consistent pattern of reduced node fraction across all backbone methods, except for the LANS filter, which maintains all nodes consistently. The Marginal Likelihood (MLF) and ECM curves start at zero node fraction. This occurrence is attributed to removing all edges in some networks, consequently resulting in removing all associated nodes.

### Weight fraction

**Fig 10:**
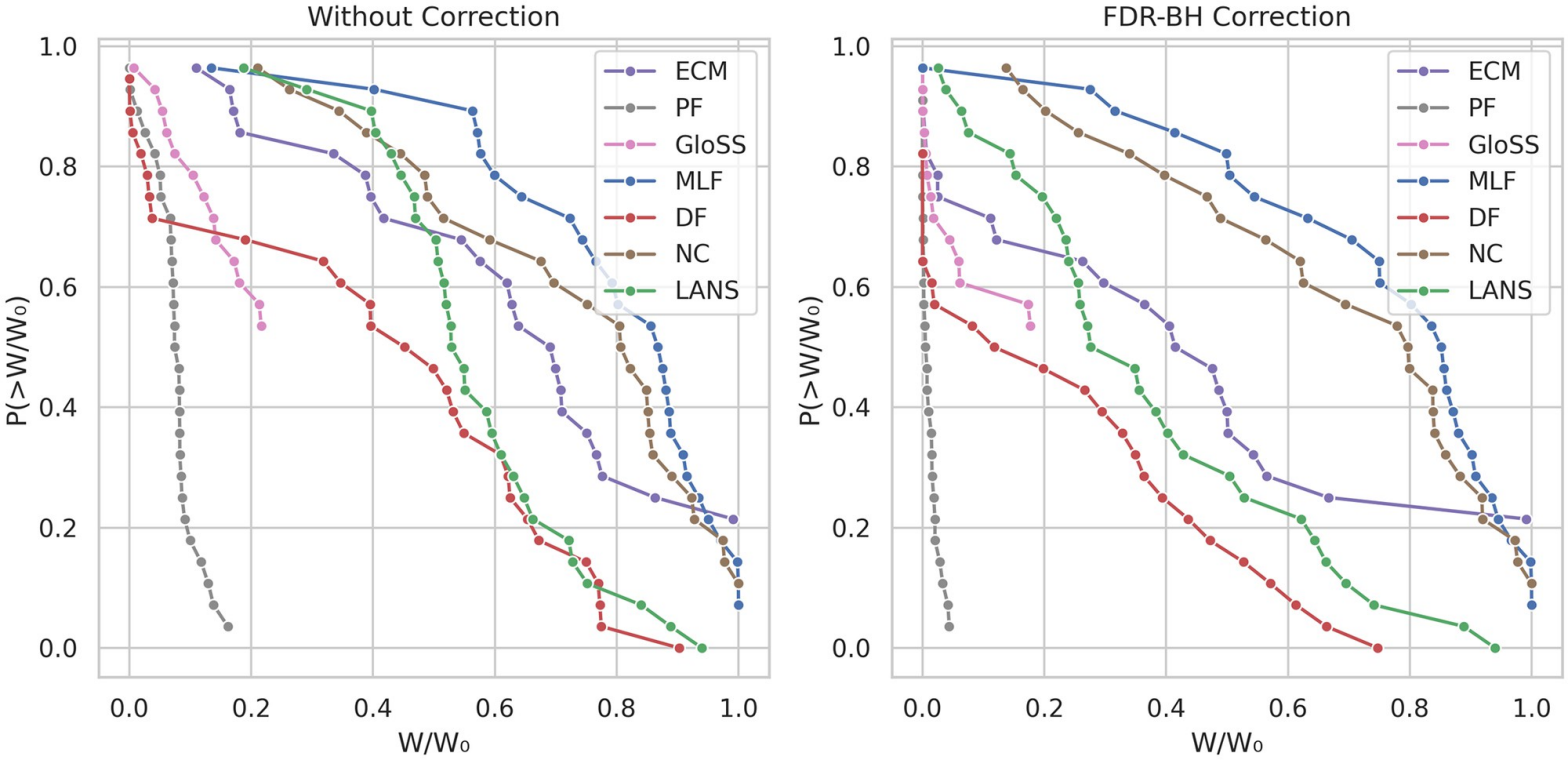
The counter cumulative distribution function of the backbones weight fraction *W*/*W*_0_ across all networks.

The statistical backbone filtering techniques diverge from global threshold methods by preserving a diversity of edge weights. However, our prior experiments have revealed that some methods preserve high edge weights while others exhibit fairness across all edge weight scales. This prompts a comparison of the fractions of retained edge weights by these methods in the extracted backbones. Fig 10 showcases the counter cumulative distribution function of the weight fraction of the backbones, both before and after FDR-BH corrections, at a significance level of *α* = 0.05.

In the left panel, two distinct behaviors emerge. The GloSS and Polya Urn (PF) filters exemplify the first behavior. Their curves commence near zero weight fraction, with tails tapering around 0.2. This is attributed to the low fraction of edges and the sparse number of backbone edges with high weights, resulting in significantly smaller weight fractions than other methods.

The second behavior is exhibited by the ECM, LANS, Noise Corrected (NC), and Marginal Likelihood (MLF) filters. Their curves start at higher weight fraction values, ranging between 0.1 and 0.2. Moreover, their tails extend to nearly reach 1, owing to the high fraction of edges and the revealed correlation between edge significance and weight. The Disparity filter (DF) falls between these behaviors. For weight fractions below 0.2, its curve aligns with that of the GloSS and Polya Urn (PF) methods, but beyond that, it aligns with the other methods. Despite filtering numerous edges akin to the Polya Urn filter (PF), it preserves a very high weight fraction. This aligns with earlier findings, indicating a higher correlation between Disparity (DF) edge p-values and edge weight.

The FDR-BH correction is applied in the right panel, and a consistent weight fraction reduction across all backbone methods is observed. Notably, despite heavy filtering by the Disparity filter (DF) and subsequent correction, high-weight fractions are still persistent, unlike similar techniques such as Polya Urn (PF) and GloSS filters, which heavily prune the network. The Marginal Likelihood (MLF) and ECM curves begin at zero weight fraction, due to the removal of all edges in some networks, resulting in the removal of associated nodes.

### Weight entropy

**Fig 11:**
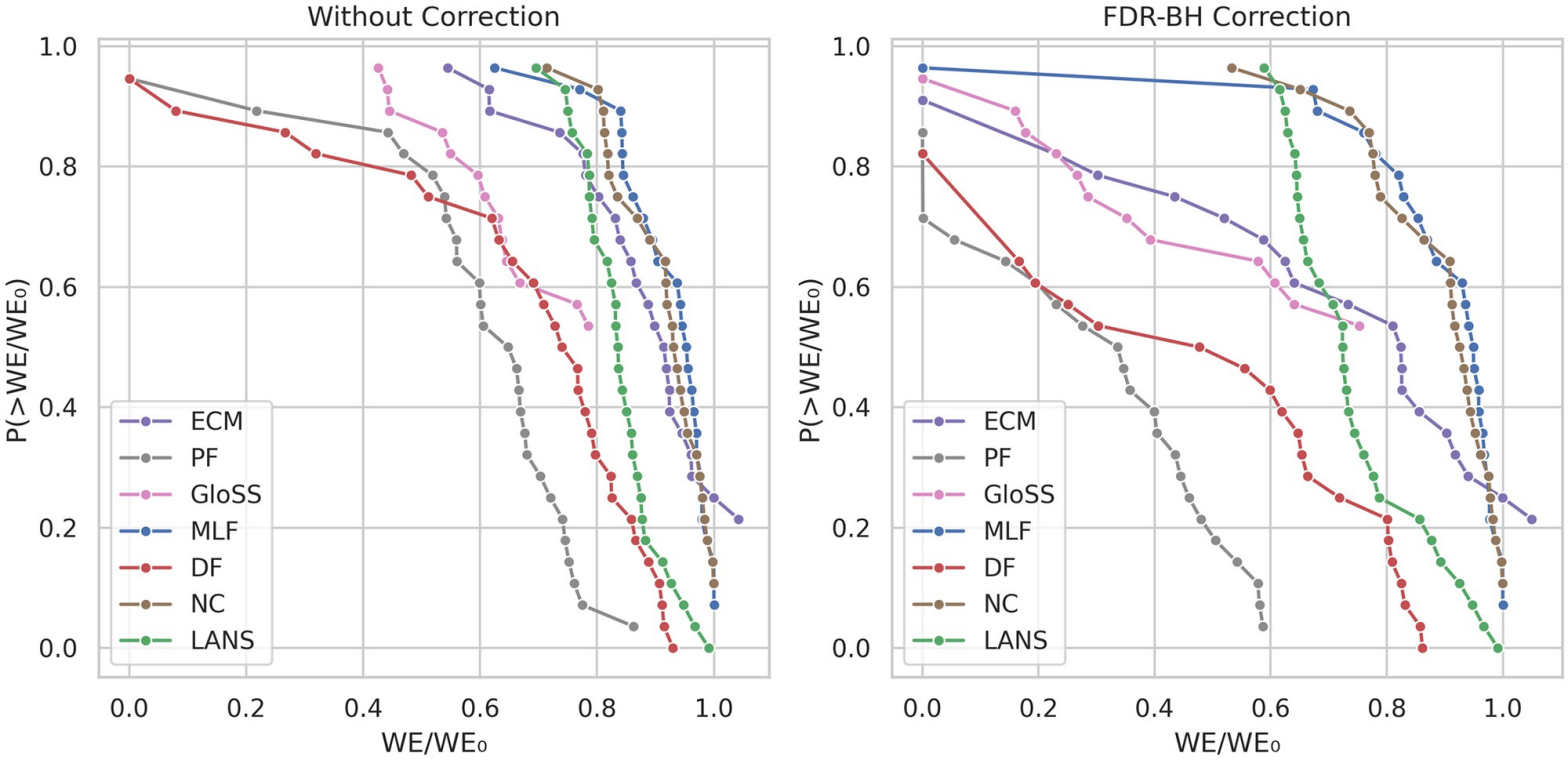
The counter cumulative distribution function of the backbones normalized weight entropy *WE*/*WE*_0_ across all networks.

The weight fraction assesses the total edge weight in the backbone. However, it does not quantify the diversity of the edge weights. Shannon entropy measures the disorder or randomness in the distribution of edge weights within the backbone, indicating how evenly or unevenly weights are distributed among edges. Higher entropy signifies greater disorder in the distribution. Fig 11 showcases the counter cumulative distribution function of the normalized weight entropy of the backbones, both before and after FDR-BH corrections, at a significance level of *α* = 0.05.

The left panel shows that some methods exhibit high normalized weight entropy. For instance, the Marginal Likelihood (MLF), Noise Corrected (NC), LANS, and ECM filters have higher normalized weight entropy values. Indeed, approximately 80% of the extracted backbones have values between 0.8 and 1, indicating the ability of these methods to preserve backbones with weight entropy closely resembling that of the original network.

Conversely, the GloSS, Disparity (DF), and Polya Urn (PF) filters display lower normalized weight entropy values. In particular, the Polya Urn filter (PL) exhibits the lowest values among all the methods. It suggests that these methods filter specific weight scales more than others, resulting in a more concentrated or less diverse distribution of edge weights.

A consistent reduction in normalized weight entropy across all backbone methods occurs in the right panel when the FDR-BH correction is applied. This reduction is particularly noticeable in the Polya Urn (PF), Disparity (DF), GloSS, and ECM filters. This indicates that false positive edge weights are not distributed evenly across all scales, leading to a disturbance in weight distribution by disproportionately removing certain weight scales over others.

### Reachability

**Fig 12:**
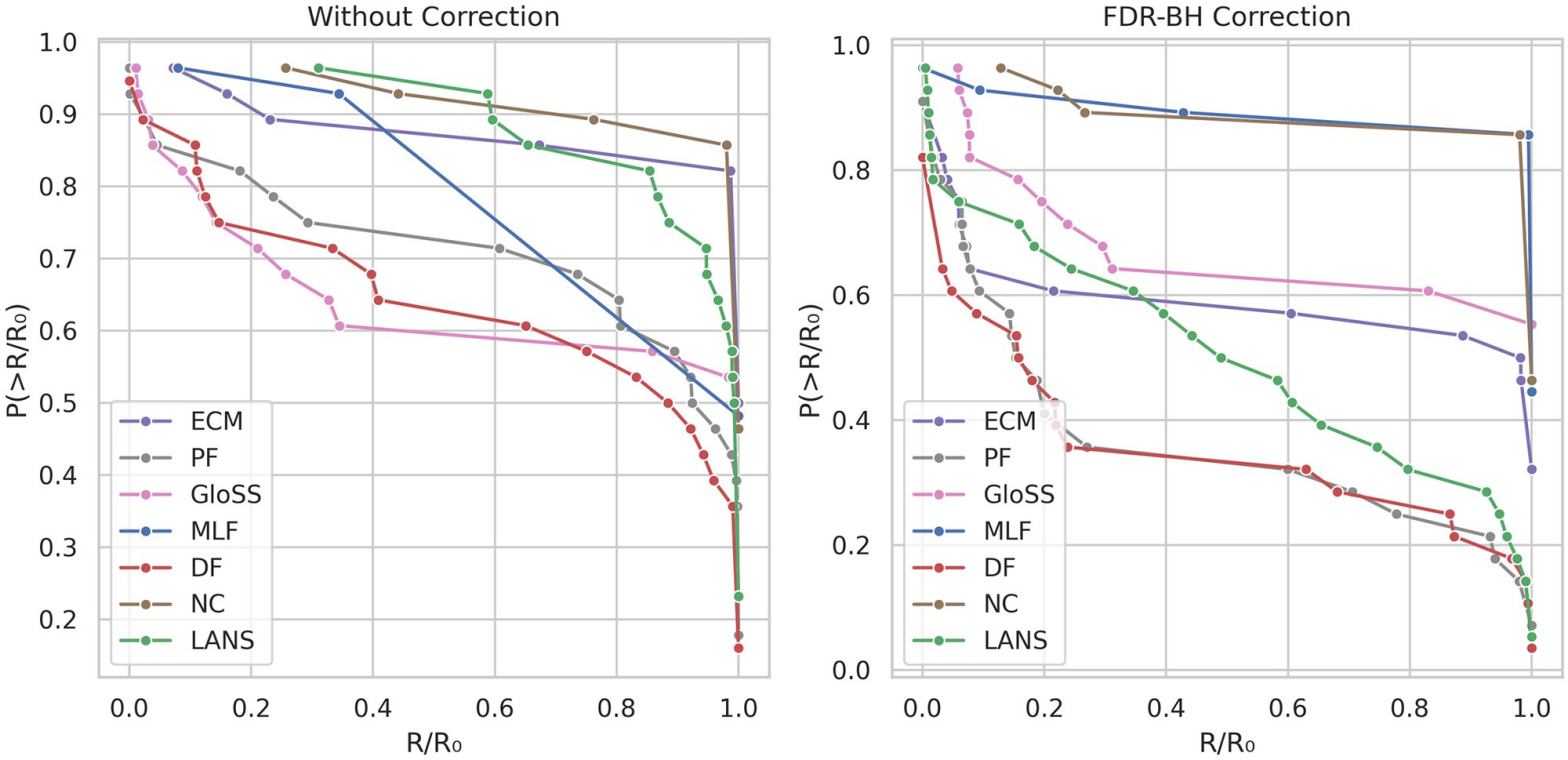
The counter cumulative distribution function of the backbones reachability *R*/*R*_0_ across all networks.

Filtering the network can potentially disrupt the connections within the network, leading to a backbone composed of multiple components. Therefore, it is crucial to evaluate backbone extraction techniques by comparing the reachability of nodes in the extracted backbones. Reachability measures the connectivity between any pair of nodes within a network. Fig 12 displays the counter cumulative distribution function of the reachability of the backbones, both before and after FDR-BH corrections, at a significance level of *α* = 0.05.

In the left panel, two distinct behaviors are apparent. The first is exemplified by the ECM, Marginal Likelihood (MLF), and Noise Corrected (NC), and ECM filters. The reachability of their backbone networks remains consistently high across all networks, hovering around 1. There are few exceptions for smaller networks whose backbone’s reachability is low, indicating these methods’ ability to filter networks while maintaining node connectedness.

The second behavior is demonstrated by the Polya Urn (PF), Disparity (DF), and GloSS filters, which exhibit varying reachability values depending on the network. This suggests that these methods may produce backbones with either a single component or multiple disconnected components, depending on the specific network characteristics. The LANS filter typically falls between these two behaviors, generally maintaining high reachability in most networks, though not always reaching a value of one.

In the right panel, after applying the FDR-BH correction, there is a consistent reduction in reachability across all backbone extraction methods. Notably, all methods begin to extract backbones with multiple components. However, the Marginal Likelihood (MLF) and Noise Corrected (NC) filters remain at the forefront, still primarily producing backbones with a single component.

### Number of components

**Fig 13:**
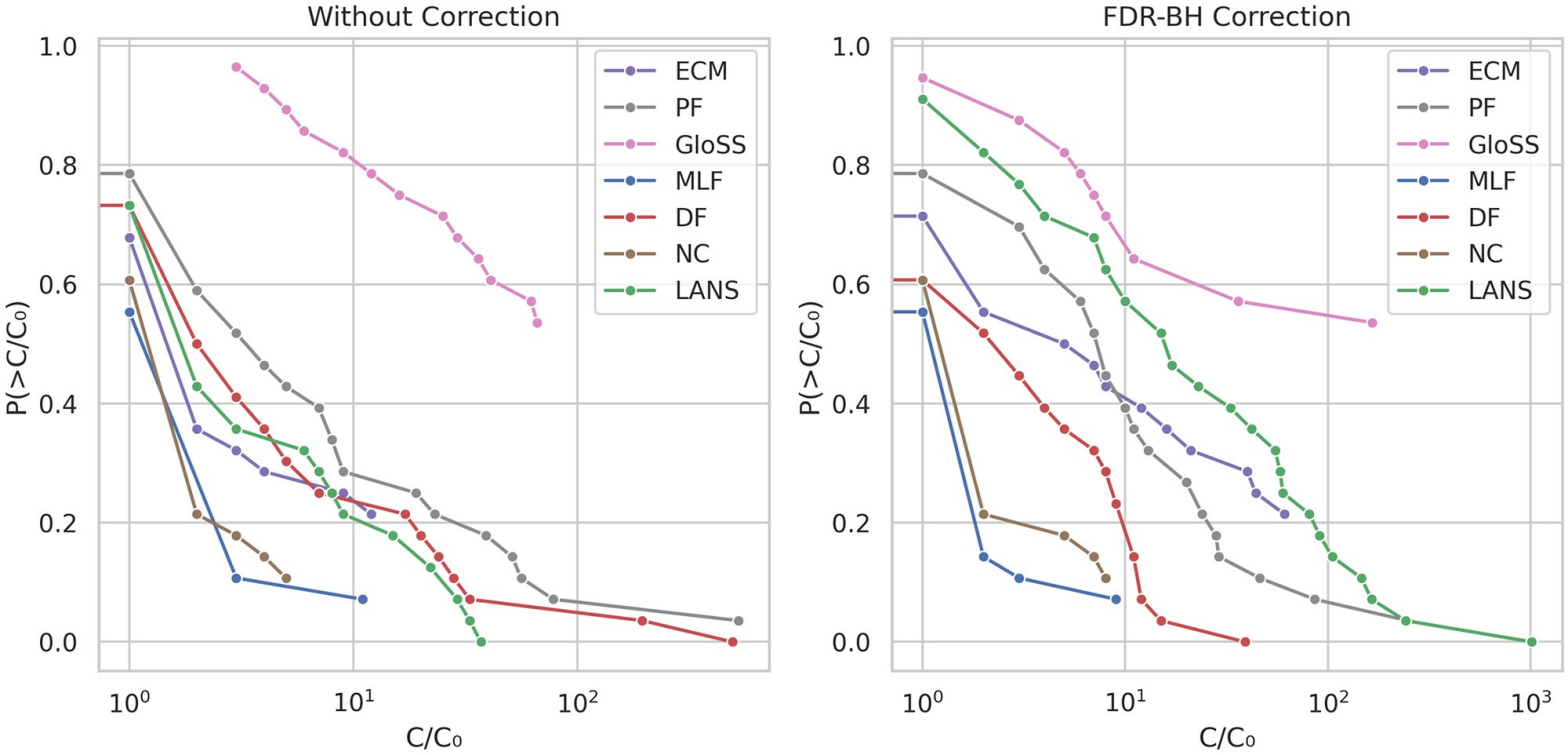
The counter cumulative distribution function of the number of components *C*/*C*_0_ in the backbones across all networks.

Low reachability values indicate network disconnection but do not elucidate how the network is fragmented. Hence, we examine the number of components in the extracted backbones to understand their connectivity better. Fig 13 presents the counter cumulative distribution function of the number of components of the backbones in logarithmic scale, both before and after FDR-BH corrections, at a significance level of *α* = 0.05.

In the left panel, the Marginal Likelihood (MLF) and Noise Corrected (NC), and ECM filters consistently yield a single component, barring a few outliers observed in small-sized networks. Conversely, the backbones produced by other methods exhibit multiple components, with some networks reaching over 100 components. Notably, the GloSS filter never generates a single-component backbone.

After applying the FDR-BH correction in the right panel, we observe two distinct behaviors. The first, exemplified by the Marginal Likelihood (MLF) and Disparity (DF) filters, shows that the correction removes edges from small components, decreasing the total number of components in the backbones. In contrast, the other methods exhibit the second behavior, where the correction leads to further fragmentation of the network, thus increasing the total number of components in the network.

### Transitivity

Transitivity in networks refers to the tendency for nodes with a mutual connection to also be connected. This property is crucial as it often leads to the formation of clusters or communities within networks, facilitating efficient information flow, collaboration, and the emergence of collective behaviors. Filtering edges may disrupt these cycles, consequently breaking the communities and hindering efficient information flow in the extracted backbones. Thus, analyzing transitivity is essential for understanding the interconnectedness among local clusters or triangles within the extracted backbones.

**Fig 14:**
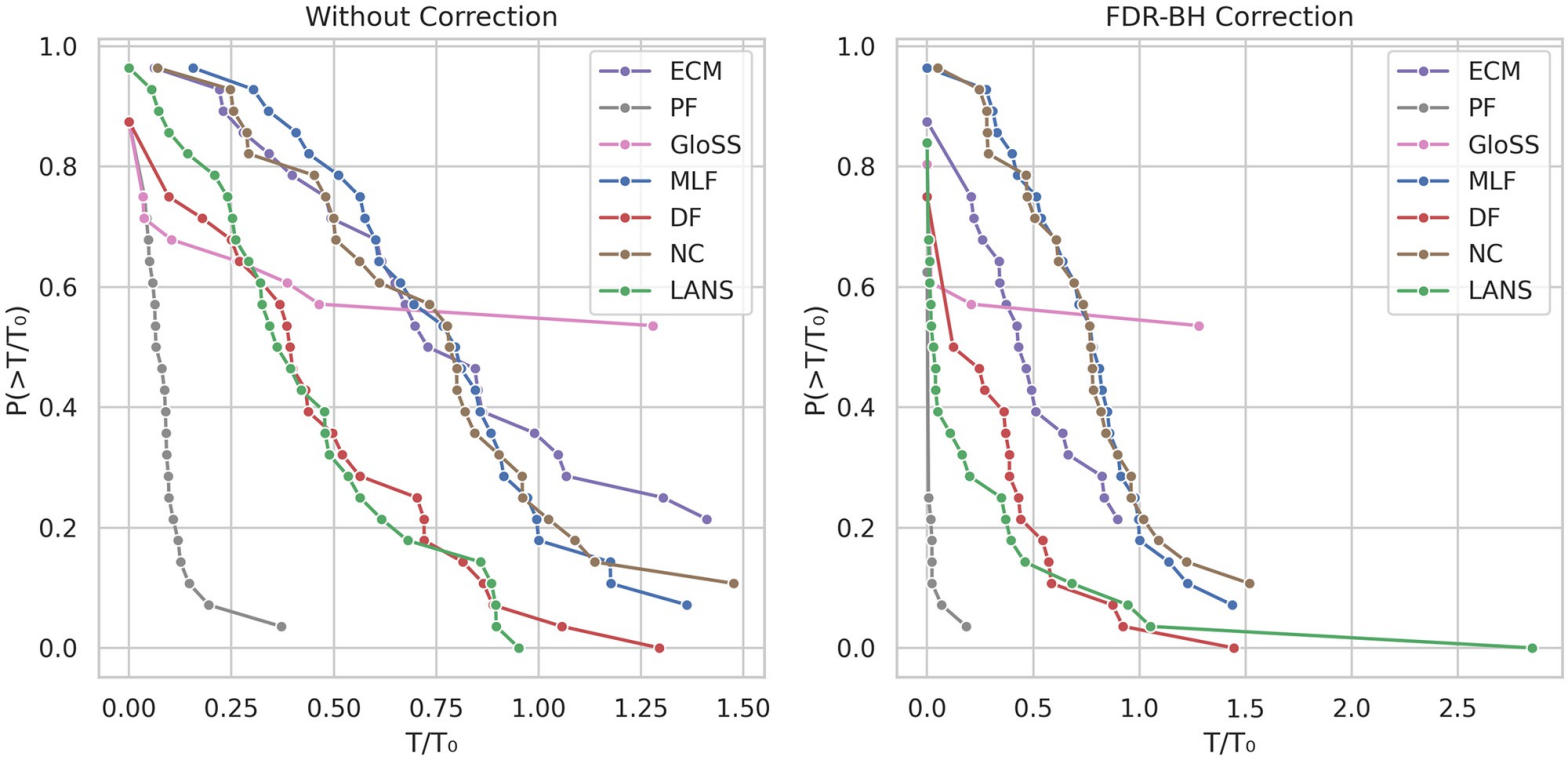
The counter cumulative distribution function of backbones normalized transitivity *T*/*T*_0_ across all networks.

Fig 14 illustrates the counter cumulative distribution function of the normalized transitivity of the backbones, both before and after FDR-BH corrections, at a significance level of *α* = 0.05.

In the left panel, the Polya Urn (PF) filter consistently exhibits uniform behavior across all networks by significantly reducing the original network transitivity. This suggests that the information flow is greatly affected due to the disruption of network triangles.

In contrast, the other methods lack consistent behavior, either decreasing or increasing transitivity depending on the network. It’s worth noting that the increase in normalized transitivity in certain networks is attributed to the preservation of cycles and the disruption of triads in the network.

The right panel illustrates the results with the FDR-BH correction. There is a consistent reduction in the normalized transitivity across all backbone extraction methods. This indicates that removing false positive edges does not alter the methods’ behavior regarding backbone transitivity.

## Comparing backbone distribution

In the previous experiment, we explored the global characteristics of backbones and identified attributes associated with each extraction method. Now, we apply a similar approach, focusing on distributions, particularly in weight and degree.

The process begins with extracting the backbones for a significance level *α* = 0.05. Next, the Kolmogorov-Smirnov statistic is calculated for each backbone distribution compared to the original network. Methods are ranked based on the KS statistic values, with the rank reflecting how closely each method aligns with the original network. Finally, the results are summarized in a boxplot. For the detailed procedure, we refer to Methods.

### Weight distribution

The comparison of the weight distribution of the filtered network backbone is crucial in evaluating the effectiveness of the filtering technique in retaining different weight scales. The weight scales in a network reflect the connections and relationships between the nodes, and a suitable filtering technique should preserve different scales of these weights, thus preserving the original weight distribution. This comparison allows us to identify which filtering methods best maintain the original network weight distribution.

**Fig 15:**
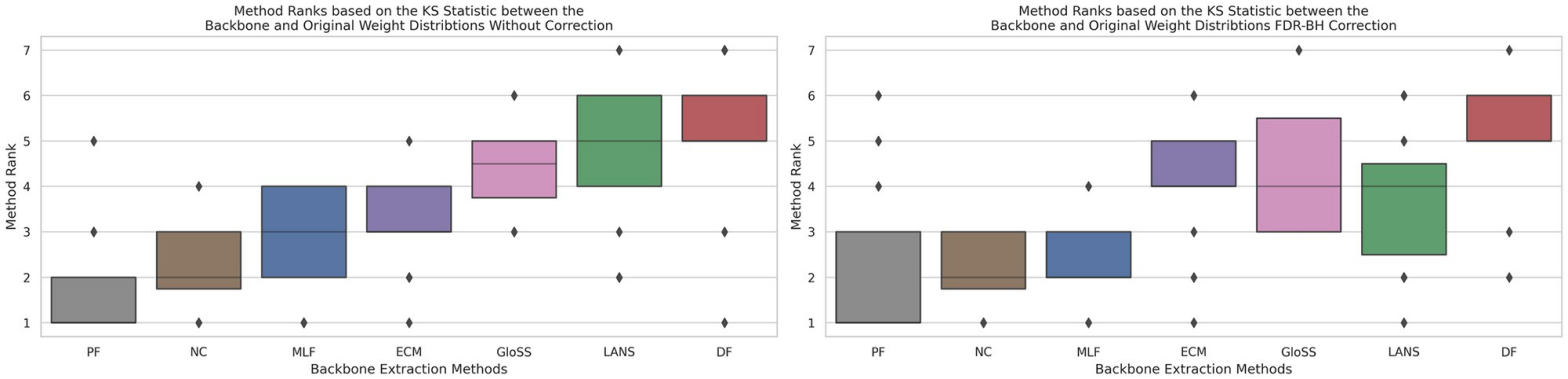
The boxplots of the backbone extraction methods rank based on the Kolmogorov-Smirnov statistic between the cumulative weight distribution of each filtering technique and the original network across all networks. Techniques include: Marginal Likelihood Filter (MLF), Disparity Filter (DF), Local Adaptive Network Sparsification (LANS), Polya Urn Filter (PF), Noise-Corrected Filter (NC), and Global Statistical Significance Filter (GloSS).

In Fig 15, the boxplots depict the rankings of backbone extraction methods based on the Kolmogorov-Smirnov statistic, measuring the disparity in cumulative weight distribution between each filtering technique and the original network across all networks. Remarkably, the Polya Urn filter consistently secures the first rank on average across all networks, extracting a backbone with a weight distribution closest to the original network compared to other methods. Following closely, the Noise Corrected filter attains the second rank on average, trailed by the Marginal Likelihood and ECM filters in the third position. Conversely, the GloSS, LANS, and Disparity filters occupy higher ranks, indicating that the weight distribution of their extracted backbones deviates significantly from that of the original network. Notably, the Disparity filter holds the last rank with a low standard deviation, emphasizing its distinctive position in terms of weight distribution dissimilarity.

The second panel reveals consistent trends, showing no significant change in the average ranks of the methods. This indicates that removing false positive edges minimally impacts the weight distribution except for a few networks.

### Degree distribution

Evaluating the degree distribution of the filtered network backbone is pivotal in assessing the filtering technique’s effectiveness in preserving diverse degrees of connectivity. Node degrees signify the connections and influence of nodes in a network, and an effective filtering method should retain various degrees, thereby conserving the original degree distribution. This comparative analysis enables the identification of filtering methods that most effectively uphold the inherent diversity of node degrees in the original network.

**Fig 16:**
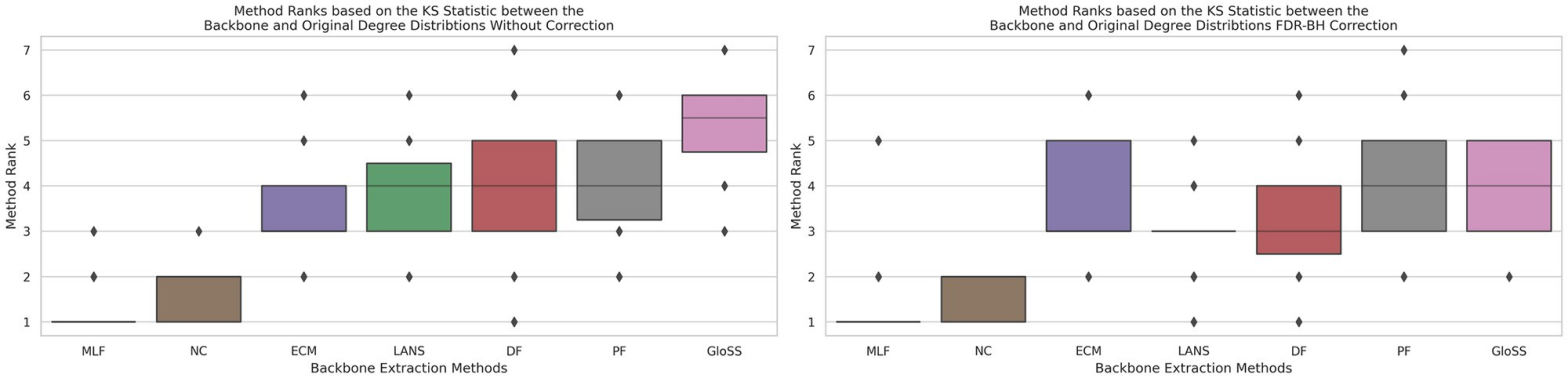
The boxplots of the backbone extraction methods rank based on the Kolmogorov-Smirnov statistic between the cumulative degree distribution of each filtering technique and the original network across all networks. Techniques include: Marginal Likelihood Filter (MLF), Disparity Filter (DF), Local Adaptive Network Sparsification (LANS), Polya Urn Filter (PF), Noise-Corrected Filter (NC), and Global Statistical Significance Filter (GloSS).

In Fig 16, the boxplots depict the rankings of backbone extraction methods based on the Kolmogorov-Smirnov statistic, measuring the disparity in cumulative degree distribution between each filtering technique and the original network across all networks. Significantly, the Noise Corrected and Marginal Likelihood filters jointly secure the top rank, extracting a backbone with a degree distribution closest to the original network, surpassing other methods. The LANS filter claims the second rank, followed by the ECM, Polya Urn, Disparity, and the GloSS filter. Notably, the backbones produced by the GloSS filter exhibit the most substantial deviation in degree distribution from the original network.

The second panel reveals consistent trends, showing no significant change in the average ranks of the methods. This indicates that removing false positive edges minimally impacts the degree distribution except for a few networks.

## Discussion

This study conducts a comparative analysis of seven statistical backbone edge filtering techniques using a dataset comprising 27 networks with diverse sizes and topological characteristics from various domains. The objective is to assess the similarity between filtering techniques and to delineate the properties associated with each method of backbone extraction.

In the initial experiment, we explore the similarity among the backbone filtering techniques. Our findings reveal that the ECM and Disparity (DF) filters yield the most distinct backbones, exhibiting minimal overlap with backbones derived from other methods. Interestingly, we uncover a hierarchical relationship among the techniques despite employing different null models and operating within distinct scopes. This hierarchy suggests a cumulative inclusion of edges as one transition from GloSS to Noise Corrected, Poly Urn, LANS, and Marginal Likelihood Backbone (*GloSS* ⊂ *NC* ⊂ *PF* ⊂ *LANS* ⊂ *MLF*), offering valuable guidance for selecting an appropriate extraction method. For example, employing the Marginal Likelihood filter could yield a backbone enriched with significant edges deviating from its null model, with a high chance of also deviating from prior null models in the hierarchy.

In the subsequent experiment, we examine the characteristics of backbone edges, examining their relationship with p-values and local edge properties such as weight, degree, and betweenness. Our analysis suggests tailoring the choice of method based on specific case studies. For instance, the Polya Urn filter (PF) emerges as optimal for maximizing fairness, exhibiting minimal correlation with edge weight, degree, and betweenness. Alternatively, the Disparity (DF) and LANS filters may serve as viable alternatives to the global threshold at the local scale, given their strong negative correlation with edge weight, indicating more significance. Moreover, the ECM filter proves valuable for identifying local connections (spoke-spoke) edges, showcasing a notable positive correlation with edge degree, which tends to undervalue such edges that typically link hubs. Notably, none of the statistical backbone extraction methods demonstrate a correlation with edge betweenness, underscoring their impartial treatment of peripheral edges.

In the third experiment, we extract backbones at a significance level of *α* = 0.05, both with and without FDR-BH correction, and evaluate their global topological properties. Our analysis reveals consistent preservation behaviors across certain properties, denoted in green, while others are network-dependent (blue) or consistently non-preservative (red). For example, the Disparity filter consistently reduces graph density significantly through heavy filtering, while the Polya Urn filter demonstrates similar behavior but fails to preserve weight entropy and transitivity. Conversely, the Marginal Likelihood and Noise Corrected filters consistently maintain entropy and network connectivity, with other properties contingent on specific network characteristics. Notably, applying FDR-BH correction identifies numerous edges as false positives, resulting in their removal primarily with the Disparity, Polya Urn, and ECM filters, while other properties remain consistent across all backbone extraction methods post-correction.

Finally, we delve into the weight and degree distributions of the extracted backbones, considering a standard significance level of *α* = 0.05. The results highlight the Polya Urn filter’s ability to capture the weight distribution of the original network, elucidating its distinctive behavior and dissimilarity observed earlier. This dissimilarity stems from its inclusive consideration of all weight scales, allowing for a more comprehensive representation of the original weight distribution.

Additionally, while previous experiments revealed a similarity between the Noise Corrected filter and the Marginal Likelihood and ECM filters, this experiment reveals subtle dissimilarities that enhance the Noise Corrected filter’s capacity to better capture the original weight distribution.

Conversely, the Disparity filter’s backbone exhibits the most considerable deviation in weight distribution from the original network.

Upon examining the degree distribution, it is evident that the Noise Corrected and Marginal Likelihood filters preserve the degree distribution compared to other methods. In contrast, the GloSS and Disparity filter’s backbones deviate the most from the original network’s degree distribution.

## Conclusion

To assist users in selecting the most suitable technique, a systematic comparison of different backbone edge filtering methods is essential. This study undertakes a comparative analysis of seven statistical backbone edge filtering techniques across 27 networks.

Correlation analysis reveals that the Disparity and LANS filters prioritize high-weighted edges, while the ECM filter assigns lower significance to edges with high degrees (edges between hubs). Properties analysis unveils three behavioral types for each topological property: consistent, inconsistent, and network-dependent behaviors. Table 1 provides a summary of the properties associated with each method. Notably, the LANS filter is the only method for preserving all nodes. In contrast, the Disparity, Polya Urn, ECM, and GloSS filters significantly decrease network size. The Marginal Likelihood, Noise Corrected, and ECM filters preserve both network connectivity and weight entropy. The LANS filter maintains the weight entropy.

**Table 1:** A recap of the characteristics of backbone filtering techniques.

Property	DF	PF	MLF	NC	ECM	GloSS	LANS
Heavy Filtering	✔	✔	✔	✔	✔	✔	✔
Node Preservative	✔	✔	✔	✔	✔	✖	✔
Entropy Preservative	✖	✖	✔	✔	✔	✖	✔
Connectivity Preservative	✔	✔	✔	✔	✔	✖	✔
Transitivity Preservative	✔	✖	✔	✔	✔	✖	✔

The symbol ✔ signifies adherence to the property, ✔ denotes network dependency, and ✖ indicates non-conformance to the property.

Distribution analysis shows that the Polya Urn preserves the original weight distribution. Following closely, the Noise Corrected filter exhibits a similar capability. the Noise Corrected and Marginal Likelihood filters excel in capturing or degree distribution.

These findings provide valuable insights for selecting the appropriate backbone extraction method based on individual method properties. Future research aims to expand this investigation to cover additional backbone extraction methods, particularly focusing on structural backbone extraction methods.

## Materials and methods

This section defines the materials and methods used in this study.

### Network properties

This subsection defines the used network properties in the evaluation process.

#### Edge degree

The Degree of an edge [32] connecting node *i* with node *j* can be defined as the product of the degrees of the incident nodes:

$$\begin{array}{c} k\left(i,j\right)=k_{i}k_{j} \end{array}$$ (1)

Thus edges associated with high degrees connect high-degree nodes in the network (hubs).

#### Edge betweenness

The Betweenness [59] of an edge *e* is the sum of the fraction of all-pairs shortest paths that pass through the edge *e*. This follows:

$$\begin{array}{c} b\left(e\right)=\sum_{s,t\in N}\frac{\sigma (s,t|e)}{\sigma (s,t)} \end{array}$$ (2)

where *N* is the set of nodes, *σ*(*s*, *t*) is the number of shortest (*s*, *t*)–paths, and *σ*(*s*, *t*|*e*) is the number of those paths passing through edge *e*.

#### Reachability

The Reachability [60] quantifies the connectivity between any pair of nodes in a network. It is defined as the fraction of node pairs that can communicate with each other. This reads:

$$\begin{array}{c} R=\frac{1}{n(n-1)}\sum_{i\neq j\in G}R_{ij}. \end{array}$$ (3)

with *n* is the number of nodes and *R*_*ij*_ = 1 if path exists between node *i* and *j* and *R*_*ij*_ = 0 otherwise. The Reachability values are in the [0, 1] range. If any pair of nodes can communicate in a network, the reachability *R* becomes 1. If *R* = 0 it means all nodes are isolated from each other.

#### Transitivity

The transitivity [61] of a network reflects how likely neighboring nodes are connected. Mathematically, it’s computed as the ratio of the number of triangles (Δ) to the number of connected triples of nodes (*τ*):

$$\begin{array}{c} T=\frac{3\times \Delta }{\tau } \end{array}$$ (4)

### Evaluation measures

This subsection defines the tools and measures used in the experiments.

#### Spearman rank correlation

The Spearman rank correlation coefficient [62] measures the strength and direction of association between two variables by assessing the monotonic relationship between their ranks rather than their actual values. It is calculated using the following:

$$\begin{array}{c} S=1-\frac{6\sum d_{i}^{2}}{n(n^{2}-1)} \end{array}$$ (5)

where *n* is the sample size, *d*_*i*_ is the difference between the ranks of corresponding values of the two variables. The Spearman rank correlation coefficient ranges from −1 to +1. A value of +1 indicates a perfect monotonic relationship where as one variable increases, the other variable also increases. A value of −1 indicates a perfect negative monotonic relationship where as one variable increases, the other variable decreases. A value of 0 indicates no monotonic relationship between the variables.

#### Overlap Coefficient

The overlap coefficient [58] or Szymkiewicz–Simpson coefficient is a similarity measure that measures the overlap between two finite sets. It is related to the Jaccard index and is defined as the size of the intersection divided by the smaller of the size of the two sets:

$$\begin{array}{c} overlap\left(X,Y\right)=\frac{\left|X\cap Y\right|}{min(|X|,|Y|)} \end{array}$$ (6)

However, in the context of this study, we calculate the overlap between the two sets based on the perspective of each set, following the formula:

$$\begin{array}{c} overlap{\left(X,Y\right)}_{X}=\frac{\left|X\cap Y\right|}{\left|X\right|} \end{array}$$ (7)

#### Shannon entropy

Shannon entropy [63] measures the average uncertainty or randomness in a probability distribution. It is calculated using the equation:

$$\begin{array}{c} H\left(X\right)=-\sum_{i}P\left(x_{i}\right){\text{log}}_{2}\left(P\left(x_{i}\right)\right) \end{array}$$ (8)

where *P*(*x*_*i*_) represents the probability of the *i*-th outcome of the random variable *X*.

#### Two-sample Kolmogorov-Smirnov

The two-sample Kolmogorov-Smirnov (KS) [64] test assesses whether two samples follow the same distribution. The KS statistic for the 2-sample test is the greatest distance between each sample’s ECDFs (Empirical Cumulative Distribution Functions). Thus, the Kolmogorov-Smirnov statistic *D* is given by:

$$\begin{array}{c} D=\text{max}|F_{\left(x\right)}-G_{\left(x\right)}| \end{array}$$ (9)

where $F_{\left(x\right)}=\frac{1}{m}\sum_{i=1}^{m}1_{\left(x_{i}\leq x\right)}$ and $G_{\left(x\right)}=\frac{1}{n}\sum_{i=1}^{n}1_{\left(y_{i}\leq y\right)}$ represent the Empirical Cumulative Distribution Functions of the two samples *X* = {*x*_1_, *x*_2_, …, *x*_*m*_} and *Y* = {*y*_1_, *y*_2_, …, *y*_*n*_}.

### Multiple correction testing

This subsection defines the two commonly used methods for multiple correction testing: the Bonferroni correction and the False Discovery Rate (FDR) correction using the Benjamini-Hochberg (BH) procedure.

#### Bonferroni correction

The Bonferroni correction [34] adjusts the significance level *α* for each particular hypothesis test by dividing it by the total number of tests *m*. This ensures that the family-wise error rate (FWER) across all tests remains at or below the desired overall significance level. Mathematically, the Bonferroni-corrected significance level (*α*_Bonferroni_) for each individual hypothesis test is calculated as:

$$\begin{array}{c} \alpha_{\text{Bonferroni}}=\frac{\alpha }{m} \end{array}$$ (10)

Alternatively, the Bonferroni-corrected p-value ((*p*_Bonferroni_)) for each hypothesis test *i* is calculated as:

$$\begin{array}{c} p_{\text{Bonferroni}}^{i}=p^{i}\times m \end{array}$$ (11)

Thus, if $p_{\text{Bonferroni}}^{i}$ is less than or equal to *α*, the null hypothesis corresponding to hypothesis test *i* is rejected.

#### False Discovery Rate (FDR) correction using Benjamini-Hochberg (BH) procedure

The FDR correction [35] controls the expected proportion of false discoveries among all rejected hypotheses. It is less conservative than the Bonferroni correction and is widely used in large-scale hypothesis testing scenarios.

The BH procedure involves ranking the individual p-values obtained from hypothesis tests in ascending order. Then, for each p-value *p*_*i*_, the critical value *q*_*i*_ is calculated as:

$$\begin{array}{c} q_{i}=\frac{i\times \alpha }{m} \end{array}$$ (12)

Where *i* is the rank of the p-value in the ordered list, *m* is the total number of hypotheses, and *α* is the desired overall significance level. Then, each individual p-value *p*_*i*_ is compared to its corresponding critical value *q*_*i*_. If *p*_*i*_ is less than or equal to *q*_*i*_, the null hypothesis corresponding to the *i*th ranked hypothesis test is rejected.

Alternatively, the adjusted p-value for the i-th ranked p-value, denoted as $p_{\text{fdrbh}}^{i}$, is calculated as:

$$\begin{array}{c} p_{\text{fdrbh}}^{i}=min1,min_{j\geq i}(m\times p(j))/j \end{array}$$ (13)

Thus, if $p_{\text{fdrbh}}^{i}$ is less than or equal to *α*, the null hypothesis corresponding to hypothesis test *i* is rejected.

### Methods

This subsection outlines the methods and procedures employed in our experiments.

#### Exploring technique similarities

The primary goal of the initial experiment is to uncover similarities among different backbone filtering techniques using two measures: the Spearman Rank correlation Coefficient (*S*) and the Overlap Coefficient (*OV*).

**Fig 17:**
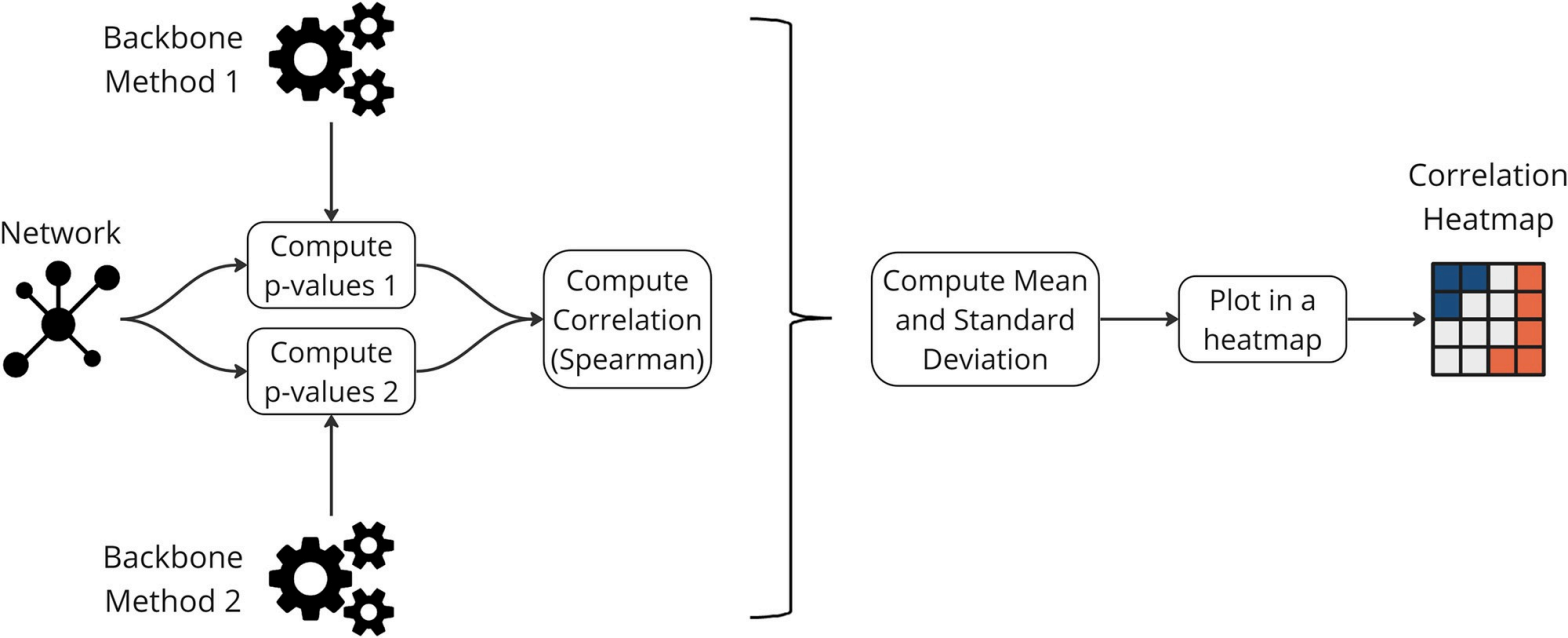
A visual representation detailing the experimental process for extracting backbones and computing the Spearman rank correlation coefficients between the p-values computed for each pair of methods.

The Spearman rank correlation explains the process in Fig 17. For each network *n*, edge p-values (*P*_*B*_) are determined for each backbone extraction method *B*.

Subsequently, Spearman rank correlation coefficients ($S_{n,B_{1},B_{2}}$) are computed for pairs of methods (*B*_1_ and *B*_2_) in each network *n*.

For each method pair (*B*_1_ and *B*_2_), the results are aggregated by calculating the mean ($\mu_{S_{B_{1},B_{2}}}$) and standard deviation ($\sigma_{S_{B_{1},B_{2}}}$) across all networks using the following formulas:

$$\begin{array}{c} \begin{array}{cc} \mu_{S_{B_{1},B_{2}}} & =\frac{1}{N}\sum_{n=1}^{N}S_{n,B_{1},B_{2}}\\ \sigma_{S_{B_{1},B_{2}}} & =\sqrt{\frac{\sum_{n=1}^{N}{\left(S_{n,B_{1},B_{2}}-\mu_{S_{B_{1},B_{2}}}\right)}^{2}}{N}} \end{array} \end{array}$$ (14)

Finally, the results are visually presented in a heatmap.

**Fig 18:**
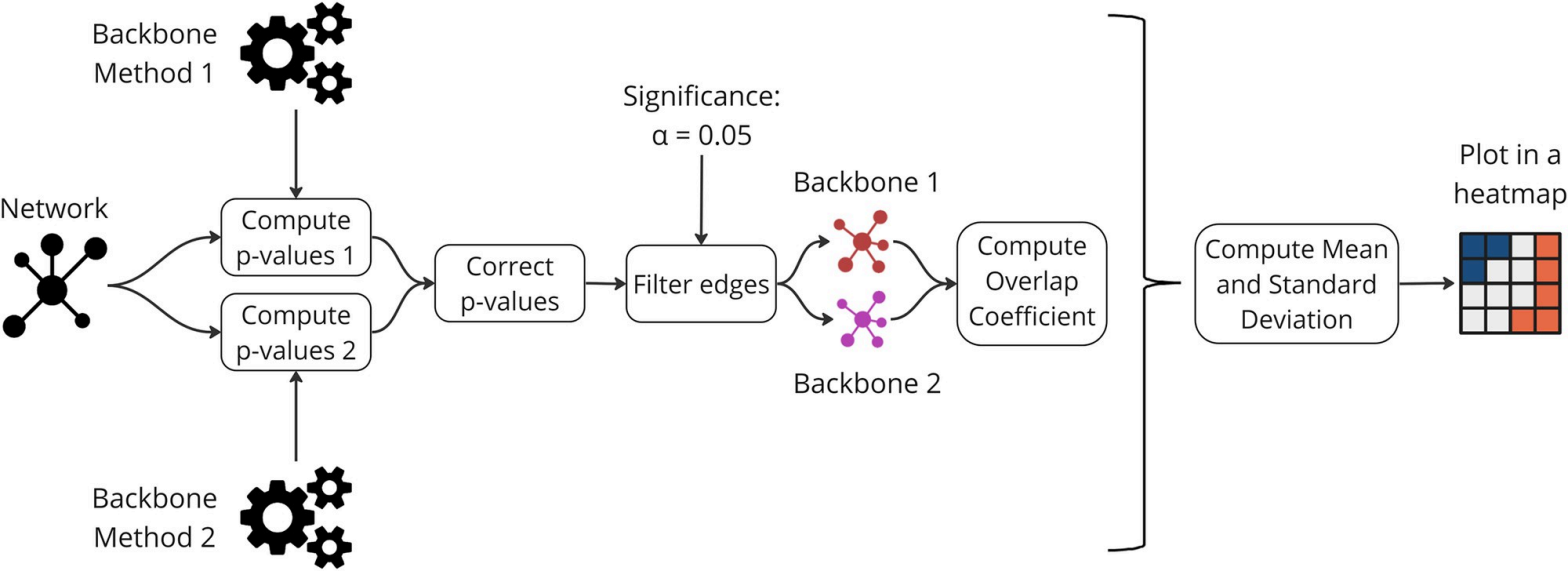
A visual representation detailing the experimental process for extracting backbones and computing the Jaccard similarity between the sets of top-scoring edges identified by the respective methods.

Fig 18 illustrates the second procedure using the Overlap Coefficient. Initially, for each network *n*, edge p-values (*P*_*B*_) are computed for each backbone extraction method *B*. Then for a significance level *α* = 0.05 we correct the edge p-values using the FDR-BH correction. Next, the backbones are extracted by removing all edges with p-values greater than the significance level *α*.

Subsequently, Overlap coefficient ($OV_{n,B_{1},B_{2}}$) are computed for pairs of methods (*B*_1_ and *B*_2_) in each network *n* using the set of edges (*E*_1_) and (*E*_2_) of each backbone.

For each method pair (*B*_1_ and *B*_2_), the results are aggregated by calculating the mean ($\mu_{OV_{B_{1},B_{2}}}$) and standard deviation ($\sigma_{OV_{B_{1},B_{2}}}$) across all networks using the following formulas:

$$\begin{array}{c} \begin{array}{cc} \mu_{OV_{B_{1},B_{2}}} & =\frac{1}{N}\sum_{n=1}^{N}OV_{n,B_{1},B_{2}}\\ \sigma_{OV_{B_{1},B_{2}}} & =\sqrt{\frac{\sum_{n=1}^{N}{\left(OV_{n,B_{1},B_{2}}-\mu_{OV_{B_{1},B_{2}}}\right)}^{2}}{N}} \end{array} \end{array}$$ (15)

Finally, the results are visually presented in a heatmap.

#### Exploring the relation between the backbone extraction methods and local network properties

This experiment explores the relationship between p-values and local edge properties, such as weight, degree, and betweenness, using the Spearman Rank Correlation coefficient to identify potential associations.

The analysis aims to uncover how the statistical significance of edges relates to specific network attributes, providing insights into the behavior and nature of the selected backbone edges. By investigating these associations, we can determine whether certain edge properties are more likely to be retained or discarded during the filtering process, thus offering a nuanced understanding of the decision-making mechanisms employed by filtering techniques.

**Fig 19:**
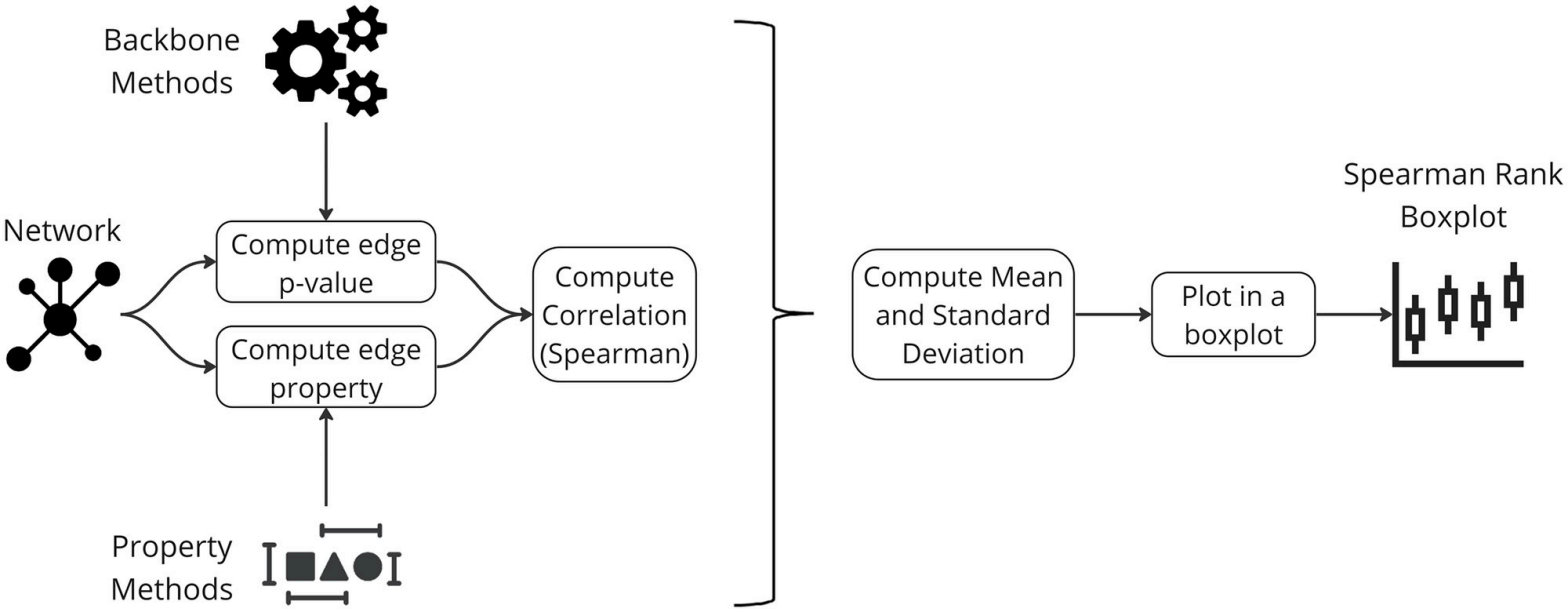
A visual representation detailing the experimental process for extracting backbones and computing the Spearman rank correlation coefficient between the edge p-values and edge properties.

In the experimental process illustrated in Fig 19, for each network *n*, we initiate by calculating the edge p-values *P*_*B*_ for each backbone extraction method *B*.

Subsequently, for each property *Y*, we compute the edge property *Y* for every edge. Following this, Spearman Rank correlation coefficients *S*_*P*_*B*_, *Y*_ are determined between the edge p-values *P*_*B*_ and each edge property *Y*.

For each edge property *Y*, a boxplot is generated to visualize the Spearman Rank correlation values for each method across all networks.

#### Exploring the backbones global properties

In this experiment, our focus shifts to examining the global characteristics of extracted backbones using various techniques. The aim is to understand how these methods impact the topological attributes of networks.

**Fig 20:**
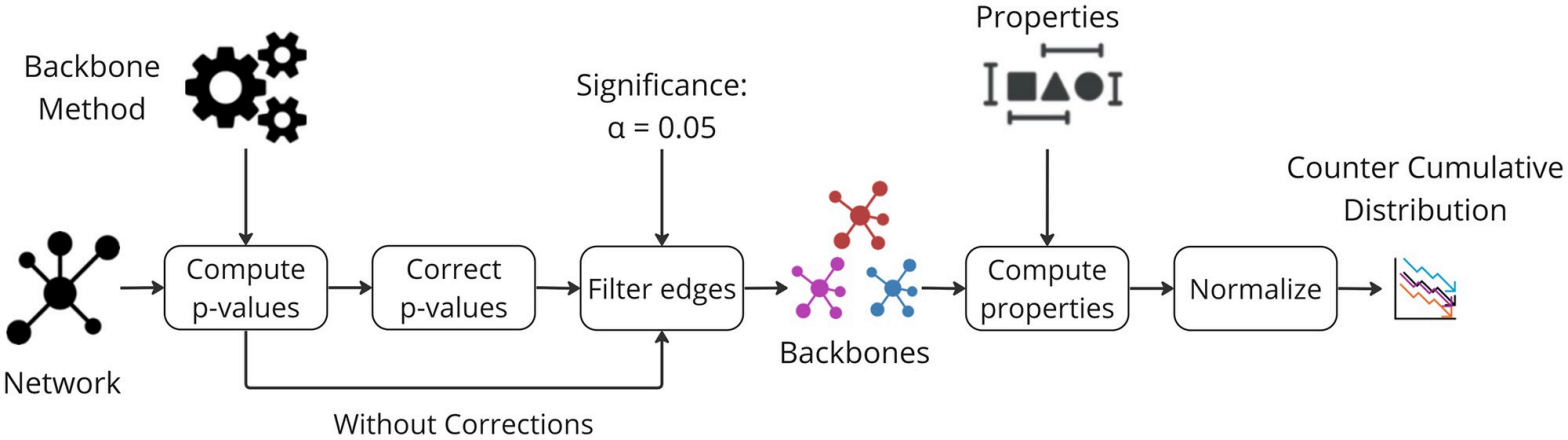
A visual representation detailing the experimental process for extracting backbones and calculating their topological properties.

The experimental procedure, illustrated in Fig 20, begins with the computation of edge p-values *P*_*B*_ using each backbone extraction method *B*.

Subsequently, a standard significance level, denoted as *α* = 0.05, is employed to filter the edges. The p-values are corrected in case of using FDR-BH or left without correction. The backbones are extracted by applying this threshold, removing all edges with p-values higher than *α*.

Following this, we calculate each topological property *V*_*Y*,*B*_ for the backbone *B* and normalize this value by the corresponding value of the original network using the following equation

$$\begin{array}{c} VN_{Y,B}=\frac{V_{Y,B}}{V_{Y,O}} \end{array}$$ (16)

The results are visualized by plotting the counter-cumulative distribution of the property values across all networks for each method.

It is crucial to note that not all methods extract a backbone. Some either preserved all the edges or removed all of them. Additionally, there were instances where the algorithm did not work on specific networks. The percentage of which the methods extracted a backbone for each method is detailed in Table 2.

**Table 2:** The percentage of extracting the backbones using each method.

	MLF	NC	ECM	GloSS	PF	DF	LANS
Without Correction	93	89	78	44	93	96	100
FDR-BH Correction	89	89	67	44	85	70	100

#### Exploring the backbones distributions

In the preceding experiment, we delved into the overarching characteristics of the backbones and unveiled the attributes associated with each backbone extraction method. In this context, we replicate the same approach, this time focusing on distributions, specifically those related to weight and degree.

**Fig 21:**
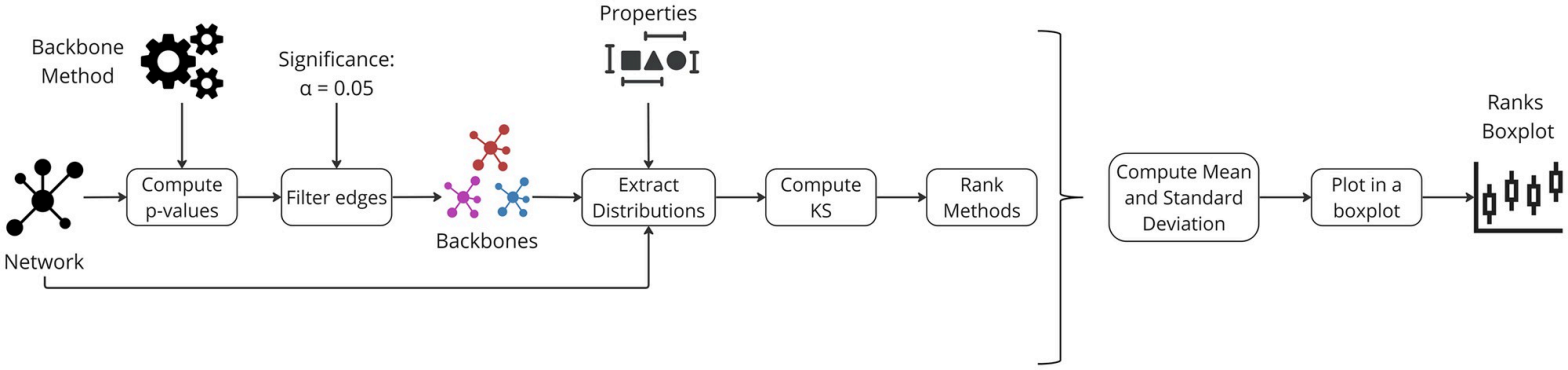
A visual representation detailing the experimental process of backbone extraction and the computation of the Kolmogorov-Smirnov (KS) statistic to compare distributions between the extracted backbone and the original network.

The experimental process, as depicted in Fig 21, initiates with the computation of edge p-values using each method.

Subsequently, a standard significance level, denoted as *α* = 0.05, is employed to filter the edges. The backbones are extracted by applying this threshold, removing all edges with p-values higher than *α*.

Then we extract the weight and degree values of both the backbones (*D*_*w*,*B*_ and *D*_*d*,*B*_) and the original network (*D*_*w*,*O*_ and *D*_*d*,*O*_).

Next, the Kolmogorov-Smirnov statistic ($KS_{D_{w,B},D_{w,O}}$ and $KS_{D_{d,B},D_{d,O}}$) is computed for each backbone distribution in comparison to the original network.

The methods are then ranked based on the KS statistic values, with a rank of 1 given to the method that is closest to the original network and 7 given to the method that is furthest.

Finally, we present the results in a boxplot. It’s noteworthy that the experiment was conducted using networks with at least 1000 edges.

## Supporting information

S1 Appendix (PDF)
