# Supplementary material for: Backbone extraction through statistical edge filtering: A comparative study
Source: PLoS One. 2025 Jan 3;20(1):e0316141. doi: 10.1371/journal.pone.0316141 (PMC11698430; doi:10.1371/journal.pone.0316141)
Supplement: S1 Appendix — (PDF) [file pone.0316141.s001.pdf]

S1 Appendix

The experiments involve 27 real-world networks spanning biological, economic, infrastructural, and offline/online social networks. S1 Table reports their basic topological features. Note that prior to analysis, we ensured compliance with the terms and conditions set forth by the data provider

Biological networks: The *Budapest Connectome* network [36, 37] is a parameterizable consensus brain graph derived from connectomes of 477 people, each computed from MRI datasets of the Human Connectome Project. Nodes are brain regions, and edges are ”tracks” that run between two regions. Weights are the number of occurrences in each of the 477 individuals.

In the *Human Connectome* network [38] nodes represent brain regions and edges are pathways mapped using DSI (diffusion spectrum imaging) of a group of five human participants in a study to map the human brain. Edge weights are the average volume of cortico-cortical axonal pathways between the human brain regions of the five participants.

The *C. elegans* network [39] is the corrected version of the neuron network of adult hermaphrodite worms C. elegans, constructed from electron microscopy series, to include edges(gap junction). Edge weights correspond to the total number of EM serial sections of connectivity.

In the *Gene Interactions* network [40], nodes are genes expressed in insect intestinal cells (Drosophila). Edges represent interactions between pairs of genes and edge weights are computed by integrating the probabilities from the various different types of evidence ('evidence channels'), while correcting for the probability of randomly observing an interaction.

The *Messel Shale* [41] is a food web network of feeling links among taxa based on the 48 million years old uppermost early Eocene Messel Shale. Edge weight denotes the certainty of the edge.

In the *Florida Bay* network [42], nodes are species, and edges denote the flow of carbon between species, typically via predation but not necessarily so. Edge weights correspond to the amount of carbon transferred.

Economic networks: The *Faculty Hiring US* [43] is a network of faculty hiring for all PhD-granting US universities for all of the academia over the decade 2011–2020. Each node is a PhD-granting institution, and a directed edge indicates that a person received their Ph.D. from an institution and was tenure-track faculty at another institution during collection. Edge weight corresponds to the total count of hires of men and women.

In the *Job Mobility* network (`https://www.michelecoscia.com/?page_id=312`), nodes are occupations in the US derived from the CensusBureau's Current Population Survey (`https://www.census.gov/programs-surveys/cps.html`). Edge weights are the number of workers who changed jobs from one occupation in 2009 to another occupation in 2010.

Infrastructural networks: In The *Worldwide Air Transportation* [44, 45] and *Openflights* [46] nodes are world airports. The former edges are direct flights connecting two airports, and edge weights represent the number of flights offered by different companies flying between 17 May and 22 May 2018. While for the latter edges are routes between two non-US-based airports downloaded from Openflights (`https://openflights.org`) on August 12, 2011, and edge weights represent the number of routes between two airports.

In the *US Airports 500* network [47], nodes represent the top 500 busiest commercial airports in the United States. An edge exists between two airports if a flight was scheduled between them in 2002. The weights correspond to the number of seats available on the scheduled flights.

In *Paris Bus* and *Paris Rail* [48] nodes represent bus and rail stops. An edge exists if a route connects two stops. The weights correspond to the number of vehicles that have traveled between two stops within a time interval.

In the *GLSN 2015* [49] nodes represents international ports. An edge exists if two ports are connected by the same service route from a dataset of 1316 international liner shipping service routes in 2015. Edge weights represent total traffic capacity (measured in TEU).

Offline social networks: In the *Karate Club* network [50], nodes are individuals from Zachary's karate club. An edge exists between two nodes if the corresponding individuals are seen together outside the normal club activities. Edge weights represent the number of such occurrences.

In the *Madrid Train Bombing Terrorists* network [51], nodes represent terrorists involved in the 2004 Madrid train bombing. Edges are associations among them, and edge weights denote how 'strong' a connection was. This includes friendship and co-participating in training camps or previous attacks.

In the *Windsurfers* network [52], nodes represent windsurfers in southern California during the Fall of 1986. Edges represent interpersonal contacts among the windsurfers, and edge weights indicate the perception of social affiliations majored by the tasks in which each individual was asked to sort cards with other surfer's names in the order of closeness.

In the *American High School* network (US-HS) [53] nodes represent students, teachers, and staff from an American High School. An edge exists if two individuals met with a time resolution of approximately 20 seconds on January 14th, 2010. Edge weights correspond to the sum of contact pairs.

In the *American Middle School* network (US-MS) [54] nodes represent students of 7th and 8th grades in a suburban middle school in Utah (USA). Edge weights correspond to the sum of contact pairs during two days, November 28th and 29th, 2012.

In the *American Elementary School* (US-ES) [54] nodes represent students of Kindergarten grades (K-6) in a suburban elementary school in Utah (USA). Edge weights correspond to the sum of contact pairs during two days, January 31th and February 2nd, 2013.

In the *French Primary School* network(Fr-PS) [55] nodes represent (232 students and 10 teachers) in a primary school in Lyon, France. Edge weights correspond to the number of face-to-face contacts recorded using active RFID devices on October 1st and 2nd 2009.

In the *French High School* network (Fr-HS) [55] nodes represent 327 high school students of specific classes called "classes préparatoires" in Lycée Thiers, Marseille, France. Edge weights correspond to the number of contacts between students over 4 days in 2013.

In the *Workplace* network (Fr-Wo) [55] nodes represent 232 individuals working in one of the two office buildings of the French Institute for Public Health Surveillance (Institut de veille sanitaire; InVS), located near Paris, France. Edge weights correspond to the number of contacts between individuals over 12 days in 2015.

In the *ACM Hypertext 2009 Scientific Conference* network (It-SC) [55] nodes represent 113 individuals attending the ACM Hypertext 2009 Conference in Torino, Italy. Edge weights correspond to the number of contacts between individuals for 3 days in 2009.

In the *Geriatric Ward of French Hospital* network (Fr-Ho) [55] nodes represent 46 hospital employees and 29 patients (75 total individuals) in a short-stay geriatric unit (19 beds) of a university hospital of almost 1,000 beds, located in Lyon, France. Edge weights correspond to the number of contacts between individuals for 4 days, from December 6th to the 10th, 2010.

Online social networks: In the *Sports Cotagging* network [56], nodes represent tags from the Sports Stack-Exchange questions (`https://sports.stackexchange.com/`). An edge exists if two tags were co-tagged in a question, and edge weights indicate the number of times of co-tagging.

In the Youtube network [57] nodes represent user profiles crawled on Dec 2008 from YouTube. In *Youtube-SF*, an edge exists if two users share at least one friend.

S1 Table. Topological features of the networks. N is the number of nodes. $|E|$ is the number of edges. $< k >$ is the average degree. ρ is the density, a is the assortativity, d is the diameter, and c is the average clustering coefficient.

Type	Network	N	|E|	<k>	ρ	a	d	c
Biological	Gene Interactions	13010	2171879	333.878	0.026	0.113	8	0.306
Biological	Budapest Connectome 3	1015	120748	237.927	0.235	0.005	4	0.727
Biological	Human Connectome	998	41693	83.553	0.084	0.153	4	0.419
Biological	Messel Shale	700	6395	18.271	0.026	-0.165	6	0.104
Biological	Florida Bay	128	2075	32.422	0.255	-0.112	3	0.335
Biological	C. elegans	460	1432	6.226	0.014	0.131	34	0.194
Offline Social	US-HS	788	118291	300.231	0.381	0.054	3	0.499
Offline Social	US-MS	591	56867	192.443	0.326	0.011	2	0.39
Offline Social	US-ES	339	16546	97.617	0.289	0.13	3	0.452
Offline Social	Fr-PS	242	8317	68.736	0.285	0.118	3	0.526
Offline Social	Fr-HS	327	5818	35.584	0.109	0.033	4	0.504
Offline Social	Fr-Wo	217	4274	39.392	0.182	0.044	5	0.381
Offline Social	It-SC	113	2196	38.867	0.347	-0.123	3	0.535
Offline Social	Fr-Ho	75	1139	30.373	0.41	-0.181	3	0.64
Offline Social	Windsurfers	43	336	15.628	0.372	-0.147	3	0.653
Offline Social	Madrid Train Bombing	64	243	7.594	0.121	0.029	6	0.622
Offline Social	Karate Club	34	78	4.588	0.139	-0.476	5	0.571
Economical	Faculty Hiring US	3284	51805	31.55	0.01	-0.362	5	0.485
Economical	Job Mobility	503	26448	105.161	0.209	-0.245	4	0.636
Infrastructural	Worldwide Air Transportation	2734	16665	12.191	0.004	-0.047	12	0.464
Infrastructural	Openflights	2905	15645	10.771	0.004	0.049	14	0.456
Infrastructural	Paris Bus	10644	12309	2.313	0	0.027	159	0.005
Infrastructural	GLSN 2015	777	12000	30.888	0.04	-0.027	5	0.722
Infrastructural	US Airports 500	500	2980	11.92	0.024	-0.268	7	0.617
Infrastructural	Paris Rail	313	408	2.607	0.008	0.353	28	0.117
Online Social	Youtube-SF	13211	1940788	293.814	0.022	0.14	9	0.488
Online Social	Sports Cotagging	237	1900	16.034	0.068	-0.288	4	0.63
